# The crosslinguistic acquisition of sentence structure: Computational modeling and grammaticality judgments from adult and child speakers of English, Japanese, Hindi, Hebrew and K'iche'^☆^

**DOI:** 10.1016/j.cognition.2020.104310

**Published:** 2020-09

**Authors:** Ben Ambridge, Ramya Maitreyee, Tomoko Tatsumi, Laura Doherty, Shira Zicherman, Pedro Mateo Pedro, Colin Bannard, Soumitra Samanta, Stewart McCauley, Inbal Arnon, Dani Bekman, Amir Efrati, Ruth Berman, Bhuvana Narasimhan, Dipti Misra Sharma, Rukmini Bhaya Nair, Kumiko Fukumura, Seth Campbell, Clifton Pye, Sindy Fabiola Can Pixabaj, Mario Marroquín Pelíz, Margarita Julajuj Mendoza

**Affiliations:** a University of Liverpool, United Kingdom of Great Britain and Northern Ireland; b ESRC International Centre for Language and Communicative Development (LuCiD); c University of Iowa, United States of America; d Hebrew University of Jerusalem, Israel; e Tel Aviv University, Israel; f University of Colorado Boulder, United States of America; g International Institute of Information Technology, Hyderabad, India; h Indian Institute of Technology Delhi, India; i University of Stirling, United Kingdom of Great Britain and Northern Ireland; j Kobe University, Japan; k University of Calgary, Canada; l University of Kansas, United States of America; m Universidad del Valle de Guatemala, Guatemala

**Keywords:** Child language acquisition, Verb semantics, Preemption, Entrenchment, Causative, English, Japanese, Hindi, Hebrew, K'iche

## Abstract

This preregistered study tested three theoretical proposals for how children form productive yet restricted linguistic generalizations, avoiding errors such as *The clown laughed the man, across three age groups (5–6 years, 9–10 years, adults) and five languages (English, Japanese, Hindi, Hebrew and K'iche'). Participants rated, on a five-point scale, correct and ungrammatical sentences describing events of causation (e.g., *Someone laughed the man; Someone made the man laugh; *Someone broke the truck*; *?Someone made the truck break*). The verb-semantics hypothesis predicts that, for all languages, by-verb differences in acceptability ratings will be predicted by the extent to which the causing and caused event (e.g., amusing and laughing) merge conceptually into a single event (as rated by separate groups of adult participants). The entrenchment and preemption hypotheses predict, for all languages, that by-verb differences in acceptability ratings will be predicted by, respectively, the verb's relative overall frequency, and frequency in nearly-synonymous constructions (e.g., X made Y laugh for *Someone laughed the man). Analysis using mixed effects models revealed that entrenchment/preemption effects (which could not be distinguished due to collinearity) were observed for all age groups and all languages except K'iche', which suffered from a thin corpus and showed only preemption sporadically. All languages showed effects of event-merge semantics, except K'iche' which showed only effects of supplementary semantic predictors. We end by presenting a computational model which successfully simulates this pattern of results in a single discriminative-learning mechanism, achieving by-verb correlations of around *r* = 0.75 with human judgment data.

## 1 Introduction

Language is a quintessentially human behaviour. But just what is it that distinguishes human language from the often-rather-sophisticated communication systems of other species? A number of distinguishing features have been proposed, including recursion (e.g., Hauser, Chomsky, & Fitch, 2002) and shared intentionality (e.g., Tomasello, 2003), but perhaps the most important and widely-agreed upon feature is productivity: Only human languages allow speakers to generate utterances that are entirely novel, that have never been encountered in the history of our species, yet are readily comprehended by any member of the relevant speech community (e.g., Chomsky, 1957; Hockett, 1960).

Explaining how children acquire this ability has long been recognized as a central question in the cognitive sciences (Bowerman, 1988). However, it turns out that this problem is even more complex than it first appeared (Baker, 1979; Braine, 1971; Pinker, 1989). The difficulty is that few of the productive generalizations that children must form are truly exceptionless. Thus children must somehow learn *not* to apply a particular generalization to exception items, while – at the same time – continuing to apply this generalization to items with which it is consistent, including items for which this generalization is novel.

Consider, for example, the language of *causation*; one of the most fundamental concepts in human cognition, and one that boasts at least one dedicated grammatical structure in probably all human languages (Nedjalkov, 1969; Comrie, 1976; Comrie & Polinsky, 1993; Haspelmath, 1993; Dixon, 2000). English-speaking children must learn that many verbs (e.g., *break*, *move*, *roll*, *spin*) can be used in the *transitive-causative* construction (e.g., *The man broke*/*moved*/*rolled*/*spun the vase*), to describe a scenario in which the AGENT (*The man*) causes the PATIENT (*the vase*) to undergo some kind of ACTION/EVENT/CHANGE. In order to achieve adultlike productivity, children must apply this generalization to verbs for which it has not been witnessed. At the same time, children must *avoid* applying this generalization to exception verbs such as *laugh*, *cry*, *fall* and *disappear*, which would yield an ungrammatical utterance (e.g., *The clown laughed/*cried*/*fell*/*disappeared the man*). Instead they must learn to convey this type of meaning using the *periphrastic-causative* construction with *make* (e.g., *The clown made the man laugh*/*cry*/*fall*/*disappear*). Conversely, many English verbs (e.g., *cut*, *tie*, *sew*, *chew*) can appear in the transitive-causative (e.g., *The boy cut*/*tied*/*sewed*/*chewed the thread*) but not the periphrastic-causative (e.g., *The boy made the thread cut/*tie*/*sew*/*chew*). Still other verbs can appear in both constructions, but with an apparent preference for one or the other (e.g., *Someone broke the truck* > *?Someone made the truck break*)

This is not merely a quirk of English. Many of the world's languages (Shibatani & Pardeshi, 2002, discuss 38 examples^1^) have (at least) two causative structures, which particular verbs prefer to a greater or lesser degree: (1) a *more-transparent* structure with the verb *cause*/*make*/*do* or a morpheme that is often a historically grammaticalized form of that verb (e.g., Japanese -(*s*)*ase*), and (2) a *less-transparent* structure that marks causation more idiosyncratically (e.g., by using a form that is either indistinguishable from a non-causative or stem form, or similar to such a form, but with a vowel or consonant change that is only partially predictable). Our terms *less-* and *more-transparent* refer to the transparency of the *surface features* that mark causation (and not, for example, to the semantics or productivity of each pattern). Table 1 shows, for each of the five languages included in the present study, examples of verbs that are grammatical in one or other, or both, causative structures.

**Table 1:** *Less-transparent* and *more-transparent* causative structures across five languages. The verb is shown in bold; the more transparent causative marker is underlined. Ungrammatical forms are marked with an asterisk (*), marginal forms with a question mark (?). Verbs marked (a) are grammatical in the more-transparent causative structure but not the less-transparent causative structure. Verbs marked (b) are, broadly speaking, grammatical in both structures (though one may be preferred). Verbs marked (c) are grammatical in the less-transparent causative structure but not the more-transparent causative structure.

Language root^a^	Less-transparent causation	More-transparent causation
English (a) laugh (b) break^b^ (c) cut	transitive-causative construction *Someone **laughed** the boy Someone **broke** the truck Someone **cut** the paper	**periphrastic-causative construction** Someone **made** the boy **laugh** ?Someone **made** the truck break *Someone **made** the paper **cut**
Japanese (a) ku, ‘come’ (b) wak, ‘boil’ (c) koware, ‘break’ (c) musub, ‘tie’	**lexical, stem-change** ?Dareka ga otokonoko o **kosas-u** [Someone SUBJ boy OBJ come-NONPAST] Dareka ga oyu o **wakas-u** [Someone SUBJ water OBJ boil-NONPAST] Dareka ga torakku o **kowas-u** [Someone SUBJ truck OBJ break-NONPAST] Dareka ga kutuhimo o **musub-u** [Someone SUBJ shoelace OBJ tie-NONPAST]	***-*(*s*)*ase*** Dareka ga otokonoko o **ko-sase-ru** [Someone SUBJ boy OBJ come-CAUS-NONPAST] Dareka ga oyu o **wak-ase-ru** [Someone SUBJ water OBJ boil-CAUS-NONPAST] ?Dareka ga torakku o **koware-sase-ru** [Someone SUBJ truck OBJ break-CAUS-NONPAST] *Dareka ga kutuhimo o **musub-ase-ru** [Someone SUBJ shoelace OBJ tie-CAUS-NONPAST]
Hindi (a) hãs, ‘laugh’ (b) gir, ‘fall’ (c) TuuT, ‘break’^b^	stem-change only (“Null” class) *kisii = ne laRke = ko hããs-aa [Someone = Erg_subj_ boy = Acc_obj_ laugh.Caus-Pfv.M.Sg] *?kisii = ne laRke = ko ger-aa* [Someone = Erg_subj_ boy = Acc_obj_ fall.Caus-Pfv.M.Sg] *kisii = ne Trak = ko toR-aa* [Someone = Erg_subj_ boy = Acc_obj_ break.Caus-Pfv.M.Sg]	**(stem change) *-aa*** *kisii = ne laRke = ko hãs-aa-yaa* [Someone = Erg_subj_ boy = Acc_obj_ laugh-Caus-Pfv.M.Sg] *kisii = ne laRke = ko gir-aa-yaa* [Someone = Erg_subj_ boy = Acc_obj_ fall-Caus-Pfv.M.Sg] *kisii = ne Trak = ko TuT-aa-yaa [Someone = Erg_subj_ boy = Acc_obj_ break-Caus-Pfv.M.Sg]
Hebrew (a) c.x.k, ‘laugh’ (b) t.m.n, ‘bury’ (c) sh.b/v.r, ‘break’	pa'al (CaCaC), pi'el (CiCeC) or hitpa'el (hiCaCeC) binyan *Mishehu **caxak** et ha-yeled [Someone laugh_PAST_ OBJ DET-boy] Mishehu **taman** et ha-'ocar [Someone bury_PAST_ OBJ DET-treasure] Mishehu **shavar** et ha-masa'it [Someone break_PAST_ OBJ DET-truck]	**hif'il (hiCCiC) binyan** Mishehu **hicxik** et ha-yeled [Someone laugh_CAUS,PAST_ OBJ DET-boy] Mishehu **hitmin** et ha-'ocar [Someone bury_CAUS,PAST_ OBJ DET-treasure] *Mishehu **hishbir** et ha-masa'it [Someone break_CAUS,PAST_ OBJ DET-truck]
K'iche' (a) war, ‘sleep’ (b) tzaq, ‘drop’ (root transitive) (b) siti, ‘turn’ (derived transitive) (c) t'iis, ‘sew’ (root transitive) (c) ram, ‘cut’ (derived transitive)	**root/derived transitive**^c^ *x-0-u-war le akal le achi PAST-3sg-SUBJ-sleep DET boy DET someone **x-0-u-tzaq** le kaxa' le achi PAST-3sg-SUBJ-drop DET box DET someone **x-0-u-suti-j** le ak'al le achi PAST-3sg-SUBJ-turn DET boy DET someone **x-0-u-t'is** le atz'yaq le achi PAST-3sg-SUBJ-sew DET skirt DET someone **x-0-u-rami-j** le wuj le achi PAST-3g-SUBJ-cut-TRN DET paper DET someone	**-(i)sa-j**^c^ **x-0-u-war-tisa-j** le akal le achi PAST-3sg-SUBJ-sleep-CAUS-TRN DET boy DET someone **x-0-u-tzaq-sa-j** le kaxa' le achi PAST-3sg-SUBJ-drop-CAUS-TRN DET box DET someone **x-0-u-sutin-isa-j** le ak'al le achi PAST-3sg-SUBJ-turn-CAUS-TRN DET boy DET someone *x-0-u-t'is-isa-j le atz'yaq le achi PAST-3sg-SUBJ-sew-CAUS-TRN DET skirt DET someone * x-0-u-ramin-isa-j le wuj le achi PAST-3sg-SUBJ-cut-CAUS-TRN DET paper DET someone

a Note that the “root” is a linguistic abstraction that, for most languages, does not become a recognizable word until it is combined with obligatory morphemes marking tense, transitivity etc.

b Hindi also has a small number of intransitive verbs that do not undergo any morphological changes (stem changes or suffixation) when causativized (e.g., *badal* ‘change’, *bhar* ‘fill’). Further, the *–aa* marker is not only used to causativize intransitive verbs, but is also used with “ingesto-reflexive” transitive verbs that involve (non-)literal ingestion, such as *eat* [*a meal*], *learn* [*a lesson*], *see* [*a painting*] to express meanings such as *feed* [*someone a meal*], ‘*teach* [*someone a lesson*]’, or ‘*show* [*someone a painting*]’. These verbs, like any other (di-)transitive verb, can then “upgrade” to *–vaa* (the so-called *second causative*) when marking indirect causation. Replacing *–aa* with *–vaa* adds an additional step in the chain of causation (*uTh-aa* 'cause something to be lifted', *uTh-vaa* 'cause someone to lift something'). However, a few (di-)transitive verbs can convey indirect causation using either the second causative suffix –vaa or the direct causative suffix –aa, e.g., likh-aa/likh-vaa ‘cause somebody to write something’, kar-aa/kar-vaa ‘cause somebody to do something’. And some intransitive (unergative) verbs take the –vaa suffix directly, e.g., gaa ‘sing’→ga-vaa ‘cause somebody to sing’ and reng ‘crawl’→reng-vaa ‘cause somebody to crawl’.

c Derived transitive verbs (including all *–isa* forms) always require the suffix *–j*. Root transitive verbs sometimes require the termination marker *–Vh*, but never when – as for all the sentences in the present study – followed by a direct object.

Exactly how this is accomplished depends on the language. English, as illustrated by the examples above, relies primarily on syntax. Japanese, Hindi and K'iche rely primarily on morphology, in the form of a more-transparent causative marker (*-*(*s*) *ase*, *-aa*, and *–*(*i*)*sa-j* respectively) and various types of less-transparent stem-change. For Hebrew, the root is defined as a three-consonant (C.C.C) pattern (e.g., *sh.b*/*v.r* for BREAK), which forms a verb only when it is inserted into a binyan template; in this case either the dedicated causal binyan *hiCCiC* (e.g., *hishbir*) or the appropriate general transitive binyan: CaCaC (e.g., *shavar*) CiCeC or hiCaCeC.

The existence of a large number of languages for which speakers must learn which verbs can and cannot appear in which of two competing causative structures makes the domain of causativization an ideal test case for accounts of how children learn to appropriately restrict their linguistic generalizations. Thus the goal of the present study is to investigate whether three theoretical proposals originally developed for – and tested on – English succeed when tested on the equivalent structures across five typologically-unrelated languages: English, Japanese, Hindi, Hebrew and K'iche' (a Mayan language, spoken in Guatemala).

The ***verb-semantics*** hypothesis (Pinker, 1989; Shibatani & Pardeshi, 2002) starts from the assumption that the distinction between verbs that allow (or prefer) *less-* versus *more-transparent* causation (e.g., *break*, *move*, *roll*, *spin* vs. *laugh*, *cry*, *fall*, *disappear*) is not arbitrary, but reflects the semantics of those verbs. The most straightforward characterization is that actions of the latter type (e.g., *laugh*) “have internal causes that would make any external prodding indirect” (Pinker, 1989: 302), meaning that causation can be expressed only via a dedicated, transparent causative marker (*make*, *-*(*s*)*ase*, *-aa*, *hiCCiC* or *–isa-j*); and even this causation is often rather indirect (e.g., Bowerman, 1988:91 points out that *John made the baby stand up* could imply simply giving an order). In contrast, verbs of the former type (e.g., *break*) are more amenable to external causation, particularly direct, physical causation (Smith, 1970). Thus, for these verbs, causation does not require a dedicated surface marker (hence “less-transparent”). Because causation is inherent in the meaning of the verb itself (e.g., *break* already means ‘cause to become broken’), this meaning comes “for free” in a basic transitive sentence.

The present study tests this prediction using a crosslinguistic measure of directness of causation proposed by Shibatani and Pardeshi (2002:89).^2^ Under this proposal,

• **More-direct causation** “entails a spatio-temporal overlap of the causer's activity and the caused event, to the extent that the two relevant events are not clearly distinguishable”, and hence is associated with ***less-transparent* causative marking**.
• **Less-direct causation** entails an event in which “both the causing and the caused event enjoy some degree of autonomy…The caused event… may have its own spatial and temporal profiles distinct from those of the causing event”, and hence is associated with ***more-transparent* causative marking**.

We therefore operationalize this measure by obtaining ratings of the extent to which the causing- and caused-events associated with particular verbs are distinct.

Previous English studies of other constructions have shown that, as predicted by the verb-semantics hypothesis, participants' ratings of the extent to which verbs exhibit semantic properties relevant for particular constructions (e.g., transfer, state change) should predict the rated acceptability of these verbs in these constructions. These include recent studies of locatives (Ambridge, Pine, & Rowland, 2012), datives (Ambridge, Pine, Rowland, Freudenthal, & Chang, 2014), verbal *un*-prefixation (Ambridge, 2013; Blything, Ambridge, & Lieven, 2014) and various constructions (Ambridge et al., 2015). All of these studies use an acceptability judgment task which, unlike elicited production, yields a graded measure of sentence acceptability, even for adults (Ambridge, 2017).

Under the ***entrenchment*** hypothesis^3^ (Braine & Brooks, 1995), repeated occurrences of a particular verb root (e.g., *laugh*) contribute to an ever-strengthening probabilistic inference that it cannot be used grammatically in structures in which it has not yet appeared (e.g., *The clown laughed the man; the transitive-causative); a kind of rational Bayesian inference from absence (e.g., Hsu, Horng, Griffiths, & Chater, 2017). Intuitively, one way to interpret entrenchment is the inference that “given how often I've heard this verb root in general, if it were permitted in this structure, I would have heard it by now”. This account predicts a negative correlation between the acceptability of a particular error (e.g., *The clown laughed the man) and the overall corpus frequency of the relevant verb root, regardless of the structure in which it occurs; a prediction supported, for English, by the corpus-judgment study of Stefanowitsch (2008).

Under the ***preemption*** hypothesis (Goldberg, 1995), the use of a particular verb in a particular target structure (e.g., *laugh* in the less-transparent structure, as in *Someone laughed the boy) is deemed increasingly ungrammatical on the basis of occurrences of this verb in a nearly-synonymous competing structure (e.g., the more-transparent structure, as in *X made Y laugh*). This account predicts a negative correlation between the acceptability of a particular error (e.g., *The clown laughed the man) and the corpus frequency of the relevant verb root in a competing structure (e.g., *X made Y laugh*); a prediction supported, for English, by the corpus and judgment studies of Goldberg (2011) and Robenalt and Goldberg, 2015, Robenalt and Goldberg, 2016.

The clearest support for the verb-semantics, entrenchment and preemption hypotheses comes from a recent reanalysis of five judgment studies (Ambridge, Barak, Wonnacott, Bannard, & Sala, 2018). In general, this reanalysis found evidence for all three effects, for all construction types, for all age groups studied (5–6 year olds, 9–10 year olds and adults). Again, however, all of these studies were of English. This is particularly problematic given that, typologically, English is rather unusual in making little use of overt morphology when marking causativity.

The goal of the present study is therefore to use the phenomenon of causative marking as a way of testing the verb-semantics, entrenchment and preemption hypotheses cross-linguistically, in English, Japanese, Hindi, Hebrew and K'iche'. These languages were chosen because they are typologically unrelated, and between them exemplify most of the different ways that languages mark causation: lexically (often with a vowel/consonant change), morphologically and syntactically (see Table 1). Most crucially, for our purposes, each exhibits two different causative structures, corresponding to *less-transparent* and *more-transparent* causation, neither of which can be applied, yielding a fully grammatical utterance, to all verbs. Thus each of these systems constitutes a suitable test case for the research question outlined at the start of this paper: how children learn *not* to apply a particular generalization to exception items, while retaining a productive generalization.

Our ultimate goal (See Section 3.7) is to build a crosslinguistically-viable account under which observed effects of entrenchment, preemption and verb-semantics fall out of a single unitary learning mechanism. First, however, it is necessary to build up a picture of the conditions under which each effect does and does not occur crosslinguistically. Thus, for each language, children (aged 5–6 and 9–10) and adults rated a *less-transparent* and *more-transparent* causative sentence for each of 60 verbs (with, in most cases, one or other form hypothesized to be less than fully acceptable). We then investigated the extent to which predictor variables instantiating the verb-semantics, entrenchment and preemption hypotheses could explain the pattern of judgments within each language. On the assumption that the learning mechanisms that yield these three effects are universal, our prediction is that all three effects will be observed for every age group in every language. Crosslinguistic differences in the magnitude of each effect cannot be predicted in advance, due to the absence of the necessary semantic and distributional information collected as part of the study. Developmental increases in the magnitude of each effect are anticipated, but again cannot be predicted in advance, as they are likely to differ depending on the detailed semantic and distributional properties of each system. Thus possible crosslinguistic and developmental effects are investigated using exploratory (unregistered) analyses.

## 2 Methods

### 2.1 Participants

Participants were, for each language, 48 children aged 5–6, 48 children aged 9–10 and 48 adult students, recruited and tested in their school or university in Liverpool (English), Tokyo (Japanese), Jabalpur (Hindi), Jerusalem (Hebrew) and Western Guatemala (K'iche'). These age groups were chosen for compatibility with the previous judgment studies listed in the Introduction. An additional 20 adult speakers of each language completed the semantic-ratings task. Participants had no known language impairments, and were first-language learners of the language in question, but invariably had had at least some exposure to English (particularly the Hindi-speakers) or Spanish (K'iche' speakers).

### 2.2 Materials

#### 2.2.1 Verbs

We selected from the Action/Process category of http://concepticon.clld.org/ (List, Cysouw, & Forkel, 2016) 60 items that (a) are lexicalized as common verbs in each of the five languages, (b) are familiar to young children, (c) span a range of direct (external) and indirect (internal) causation, and (d) can be easily depicted in animations, such that naïve participants are able to guess the intended verb (or a close synonym). This was achieved through successive rounds of piloting, using incorrect guesses to refine the animations and discard unsuitable candidate verbs. Details of the verb-guessing pilot can be found in Appendix 1.

#### 2.2.2 Sentences

For each verb, we generated a *less-transparent* and *more-transparent* causative form for each language (see https://osf.io/s3cj4/). Generation of the more-transparent form was straightforward, since – by definition – this form simply combines the verb root and the relevant causative marker or binyan (see the rightmost column of Table 1). Generation of the less-transparent form was straightforward for English, where this form is always a syntactic transitive-causative. For Hebrew, Japanese, Hindi and K'iche' we used existing literature (Berman, 1993; Shibatani & Pardeshi, 2002; Matsumoto, 2016; Bhatt & Embick, 2003; Pye, 1991) to identify sub-regularities amongst the verbs for which such a form exists in the language, and applied these sub-regularities to the remaining verbs for which no such form exists.

For each verb form (less-transparent and more-transparent), we created a sentence with *Someone* as the AGENT (CAUSER) and a plausible CAUSEE as the PATIENT (e.g., *Someone broke the truck*; *?Someone made the truck break*; *Someone laughed the boy; *Someone made the boy laugh*). It was not possible to control animacy of the causee across verbs, since some (e.g., *boil*) are natural only with a nonhuman causee (e.g., *water*), and others a human causee (e.g., *laugh*). Indeed the propensity of particular verbs to occur with animate versus inanimate causees is probably an important semantic factor that determines the relative acceptability of less-transparent and more-transparent causation; a factor intended to be captured by our semantic predictor. The same AGENT-CAUSER (always *Someone*) and the same PATIENT-CAUSEE were used across both members of each less-transparent/more-transparent verb pair, and (in translation) across all languages.

#### 2.2.3 Animations

For each pair of less-transparent/more-transparent sentences, we created, using Moho Debut 12 (http://my.smithmicro.com/anime-studio-debut.html), a single animation depicting the caused action, but not the causer or causing event. This was necessary because the precise properties of the causer and causing event influence both the semantic ratings task (see below) and the relative acceptability of *less-transparent* versus *more-transparent* causation (e.g., the relative acceptability of *Someone broke the truck* and *Someone made the truck break* differs depending on whether the truck is smashed with a hammer, is pushed beyond its limits, or breaks spontaneously due to insufficient maintenance). In order to depict the caused action but not the causer or causing event, each animation begun with the causee (e.g., a truck; water) alone onstage. The curtains then closed, and reopened to show the caused event either completed or ongoing (e.g., a broken truck; boiling water). Each animation included a suitable sound effect (e.g., breaking truck; bubbling water) occurring or beginning behind the closed curtains. Identical animations were used in the grammaticality judgment task and – without accompanying sentences – the semantic ratings task. We also created six animations for practice trials for the semantic rating task and seven for grammaticality judgment training. All animations can be viewed online at https://osf.io/pavm7/.

### 2.3 Procedure

#### 2.3.1 Grammaticality judgment task

The grammaticality judgment task used the procedure outlined in Ambridge et al. (2008: 105–107). In brief, participants select either a red counter (to indicate ungrammatical) or a green counter (to indicate grammatical), then place this counter on a five point smiley face scale ranging from sad (red) to happy (green) to indicate the degree of (un)grammaticality. The procedure is presented as a game in which the child's task is to help a toy dog (who produces the sentences via a loudspeaker) to speak English (Japanese, etc.), by providing feedback on his descriptions of the animations. Because 120 trials was considered to be too many for young children, each participant (including adults) completed half of the total, according to one of eight different counterbalance lists. Trials were presented using PsychoPy2 (Peirce, 2007). Before the judgment task, participants completed a training session during which they received feedback for seven sentences with varying degrees of acceptability (translations of those listed in Ambridge et al., 2008: 124).

#### 2.3.2 Semantic ratings tasks

The aim of this task (which was conducted with adults only) was to derive a predictor variable that instantiates the verb-semantics hypothesis outlined in Shibatani and Pardeshi (2002); that a verb's relative preference for *less-transparent* (more direct) versus *more-transparent* (less direct) causation reflects the degree to which the causing and caused event (a) merge into a single event or (b) are distinct events. Participants were given the following instructions (in translation):

You will see 60 videos in which a PERSON/THING on a stage carries out/undergoes an ACTION/EVENT/CHANGE. This ACTION/EVENT/CHANGE is caused (while the curtains are shut) by a mystery UNSEEN CAUSER. Is it a person? Is it a thing? Is it the same for each video? Who knows…

An animation was then shown, accompanied by the following text (at the top of the screen)

Here, A (THE UNSEEN-CAUSER) causes B (the PERSON/THING on the stage) to carry out/undergo an ACTION/EVENT/CHANGE. We are interested in the extent to which A causing the ACTION/EVENT/CHANGE and B undergoing the ACTION/EVENT/CHANGE are separate. Please rate the extent to which…

Displayed below the animation was a visual analogue scale with the following anchors:

(Left) B's ACTION/EVENT/CHANGE and A's causing of it are two separate events, that could happen at different times and/or in different points in space.

(Right) B's ACTION/EVENT/CHANGE and A's causing of it merge into a single event that happens at a single time and a single point in space

Trials were presented in random order, using PsychoPy2. As well as this main semantic ratings task, participants also completed three subsidiary rating tasks, each designed to tap into a particular aspect of Shibatani and Pardeshi's (2002) event-merge hypothesis: (a) **autonomy** of the causee, (b) whether the caused event **requires a causer**, and (c) whether causation is **directive** (e.g., giving an order) or **physical**. Details of these tasks can be found in Appendix 2. The four tasks were presented in randomized order.

### 2.4 Predictor variables

#### 2.4.1 Verb-semantics

The predictor variable instantiating the verb semantics hypothesis was the mean rating (across all 20 semantic raters^4^) for each verb on the event-merge semantic rating task described above, with responses scaled into Z scores.

#### 2.4.2 Preemption

Under the preemption hypothesis, the use of a particular verb in a particular target structure (e.g., *laugh* in the less-transparent structure, as in *Someone laughed the boy) is deemed increasingly ungrammatical on the basis of occurrences of this verb in a nearly-synonymous competing structure (e.g., the more-transparent structure, as in *X made Y laugh*). Thus, at first blush, the hypothesis appears simply to predict a negative correlation between the rated acceptability of a particular form (e.g., *Someone laughed the boy) and the corpus frequency of this verb in the relevant competing structure (e.g., *X made Y laugh*). However, this is an over-simplification, because it fails to consider the effect of attested corpus uses of the verb in the target structure (e.g., *Someone broke the truck*), which boost its acceptability, and mitigate against preemption (e.g., *Someone made the truck break*). This is a marginal phenomenon for verbs such as *laugh* (where a corpus instance of *X laughed Y* would constitute an occasional slip of the tongue), but not for verbs like *break* that occur to some extent in both structures (*Someone broke the truck; Someone made the truck break*). Thus any measure of preemption must factor in the frequency of the verb in both the target structure (here, the less-transparent causative) and its competitor (here, the more-transparent causative). It must also factor in the base-rate of the two competing structures: For example, based on counts in Ambridge et al., (2018) the English transitive-causative (*X VERBed Y*) is roughly 100 times more frequent than the English periphrastic-causative (*X made Y VERB*). So a hypothetical verb that occurred with equal frequency in each structure (in absolute terms) would – relative to this base rate – be showing a huge bias in favour of the periphrastic-causative.

We therefore follow Ambridge et al. (2018) in operationalizing preemption using the chi-square statistic. This represents the extent to which a particular verb's distribution between two competing structures (here, the *less-transparent* and *more-transparent* causative) differs from all other verbs in our verb set (intended to constitute a reasonable approximation of verb behaviour in the language as a whole). A detailed description of the procedure is given in Ambridge et al. (2018), and illustrated in Table 2 with an example for *giggle* (counts from Ambridge et al., 2018).

**Table 2:** Example calculation of the chi-square preemption statistic for *giggle*.

	*Less-transparent* structure (English transitive-causative, *X VERBed Y*)	*More-transparent* structure (English periphrastic-causative, *X made Y VERB*)
*Giggle*	0	7
All other verbs	468,636	3676

*Chi*^2^ = 1,387,700.

*Giggle* reverses the general trend whereby the transitive-causative vastly outnumbers the periphrastic-causative, yielding a very large chi-square value. Note that, as in Ambridge et al. (2018) the polarity of the predictor is set to positive if – relative to all other verbs in the corpus – the verb is biased in favour of the structure being rated, and negative if it is biased towards the competing structure.

Preemption is calculated in the same way for each language, though of course across different surface structures (see Table 1 and accompanying description). While the English counts reflect uses of each verb root in particular syntactic word-order constructions (transitive-causative and periphrastic-causative), the Japanese, Hindi and K'iche' counts reflect uses of each verb root in particular morphological forms (e.g., of Japanese *koware*, ‘break’, in the forms *kowas-u*, *koware-sase-ru* and equivalent forms with other tense/aspect marking). Hebrew counts reflect uses of each verb root (e.g., *sh.b*/*v.r*) in the general transitive binyan template that is appropriate for that verb CaCaC (e.g., *shavar*) CiCeC or hiCaCeC versus the dedicated causal binyan templated *hiCCiC* (e.g., *hishbir). Counts were taken from the OpenSubtitle corpus at http://opus.lingfil.uu.se/OpenSubtitles2016.php (Lison & Tiedemann, 2016), which includes very large spoken corpora for English (2.5g words), Japanese (17m), Hindi (0.6m).

For Hebrew, we had initially intended to use the OpenSubtitle corpus (44m words), but switched to Linzen's 165m word Hebrew Blog Corpus (http://tallinzen.net/resources/) because its part-of-speech tagging and morphological disambiguation allowed us to exclude other forms (e.g., noun forms) that are homographs with the target search forms. Although corpora of child-directed speech would have been more representative, even the largest available are far too small to capture uses of the target verbs in the relevant structures, which are often rather infrequent (e.g., the English periphrastic-causative). For K'iche', we created a master corpus by combining (a) Furbee-Losee's (1976) *Mayan texts I*, (b) Velleman's (2014) *K'ichee' collection of Leah Velleman* at the *Archive of the Indigenous Languages of Latin America*, (c) Matzar-González, Matzar-González, and Ajpacaja's (2001) *Story of Florentino Pedro Ajpacaja*, (d) Wick and Cochojil-Gonzalez's (1975) *Quiché Maya-English Vocabulary* (e) Wick and Cochojil-Gonzalez's (1977) *Spoken Quiché Maya*, (f) Can Pixabaj's (2010) *Documentation of Formal and Ceremonial Discourses in K'ichee'*, (g) Hernández, Ramírez, Velásquez, and Domingo's (1998) *Popol Wu*j, (h) Mondloch's (1968–1973) *K'iche' Maya Oral History Project* and (i) Pye's (1991) corpus of parent-child conversations (five dyads). However, even this combined master corpus contained very few of the target verb forms. K'iche' was included despite the unavailability of a large corpus, because it was deemed important to represent a range of languages spoken in non-WEIRD (Western, Educated, Industrialized, Rich, Democratic; Henrich, Heine, & Norenzayan, 2010) countries, none of which have large corpora.

For English and Hindi, we parsed the corpus using, respectively, spaCy (Honnibal & Johnson, 2015) and the IIIT-Hindi parser (Avinesh & Karthik, 2007), and automatically extracted (for the preemption measure) candidate more- and less-transparent causative uses of each verb. For each verb and each sentence type, we then hand coded a randomly-selected 50 sentences of each type, and pro-rated these counts to get the final estimates. Overall verb counts (for the entrenchment measure) were obtained automatically. For Japanese, all counts were obtained automatically, used a pre-parsed version of the Open Subtitles corpus (KyTea parser, Neubig, Nakata, & Mori, 2011). Hand coding was not necessary since the agglutinative nature of Japanese means that the parsed corpus contains very few ambiguous forms. For K'iche', all candidate forms of each verb – less-transparent, more-transparent and other – were automatically extracted from the master corpus, and fully hand coded. Code for the English, Hindi, Japanese and K'iche' corpus analyses can be found at https://osf.io/pavm7/. For Hebrew, counts were obtained by hand, using the online interface at http://tallinzen.net/search/.

#### 2.4.3 Entrenchment

Under the entrenchment hypothesis, repeated occurrences of a particular verb root (e.g., *laugh*) in any structure contribute to an ever-strengthening probabilistic inference that it cannot be used grammatically in structures in which it has not yet appeared (e.g., *The clown laughed the man; the transitive-causative). Again, however, it is important to factor in both (a) the frequency with which the verb root has appeared in the target structure and (b) the overall frequency of that structure in the language. Thus entrenchment was calculated in the same way as preemption, except that the counts in the right-hand column reflect uses in all non-causative structures (see Table 3). That is, although corpus uses that count towards preemption *in principle*, also count towards entrenchment, in order to minimize collinearity between the two predictors, any corpus uses already counted towards preemption were excluded when calculating the entrenchment predictor. It is important to bear in mind that this constitutes a very conservative test of entrenchment, as it tests a specific and narrow prediction of the hypothesis: that attested occurrences of a particular verb root will contribute to the perceived ungrammaticality of attested uses, *even when the two are not in competition for the same message*.

**Table 3:** Example calculation of the chi-square entrenchment statistic for *giggle* vs (a) *less-transparent* causative structure and (b) *more-transparent* causative structure.

	*Less-transparent* structure (English transitive-causative, *X VERBed Y*)	All non-causative uses
*Giggle*	0	649
All other verbs	468,636	1,908,895
*Chi*^2^ = 4,120,100		


	*More-transparent* structure (English periphrastic-causative, *X made Y VERB*)	All non-causative uses
*Giggle*	7	649
All other verbs	3676	1,908,895

*Chi*^2^ = 5,705,100.

An example is shown in Table 3 (using counts from Ambridge et al., 2018). Note that, for each verb, two different entrenchment predictors are calculated for each language: one for predicting ratings of that verb root in the *less-transparent* structure, the other for predicting ratings of that verb root in the *more-transparent* structure. For example, relative to all other verbs, *giggle* is strongly biased away from the transitive-causative (indicated by negative polarity) and strongly biased (though to a lesser degree) towards the periphrastic-causative (indicated by positive polarity).

## 3 Results

The analyses reported below (with the exception of those designated as exploratory) were pre-registered, in the form of draft Introduction and Methods sections, which included both the hypotheses to be tested and the statistical methods to be used, archived on the website of the Open Science Framework in August 2017 (https://osf.io/69ehu/). Minor changes have been made to the Introduction – mainly adding additional information requested by reviewers – but these changes are not substantive, in that no changes have been made to the hypotheses. More major changes were required with regard to the analysis strategy, however: The original preregistration stated that “Bayesian mixed effects models were fitted in R (R Core Team, 2016) by using the glimmer and map2stan functions of the rethinking package (McElreath, 2016), to pass reformatted data and lme4 syntax (Bates, Maechler, Bolker, & Walker, 2015) to the rstan package (Stan Development Team, 2015a, Stan Development Team, 2015b; Carpenter et al., 2016)”. However, observed conlinearity between the entrenchment and preemption predictors meant that the coefficients (and associated pMCMC values) would essentially be uninterpretable. Thus we decided to switch to frequentist mixed effects models, which allow for *p* values to be obtained using a leave-one-out procedure (e.g., Barr, Levy, Scheepers, & Tily, 2013). This procedure avoids the problem of collinearity, because it works by comparing a full model against a model with the predictor of interest removed (i.e., the predictor of interest is never evaluated ‘in situ’ in a model containing other predictors with which it is collinear).

Mixed effects models were fitted in R (R Core Team, 2016), using the lme4 package (Bates et al., 2015). *P* values were obtained using the lmerTest package (Kuznetsova, Brockhoff, & Christensen, 2017), which overloads the “lmer” function of lme4, adding to the summary output table *p* values calculated via Satterthwaite's degrees of freedom method. Importantly, for the present design, these *p* values are identical to those calculated using the lmerTest “drop1” function, which uses a *F* test to compare models with and without the predictor of interest. (We confirmed this by running sample models with both methods, and by asking the authors of the lmerTest package, who replied that “since one df *F*-tests are the same as *t*-tests, and since all terms are marginal to each other, the outputs from drop1 equals those from summary”; see https://github.com/runehaubo/lmerTestR/issues/33#issuecomment-602105339). This method is similar to that provided by the “drop1” function of the lme4 package itself, which also compares models with and without a predictor of interest but, according to the lmerTest manual at https://cran.r-project.org/web/packages/lmerTest/lmerTest.pdf, “compared to the likelihood ratio tests of *lme4::drop1* the F-tests and p-values oflmerTest::drop1are more accurate”.

For the main analysis (simultaneous regression), we built 30 models: one for each combination of language (English, Japanese, Hindi, Hebrew, K'iche'), age-group and sentence type (less-transparent causative/more-transparent causative). Following Ambridge et al. (2018) we also built supplementary non-partial models, each with a single predictor (to aid interpretation in cases of multicollinearity in the main analyses) and models using difference scores (preference for *more-transparent* over *less-transparent uses*), as opposed to raw sentence ratings, as the DV.

All models used (almost) maximally-specified random-effects structure (Barr et al., 2013), with random intercepts for Verb and Participant, and – except for a handful of exceptions^5^ – by-participant random slopes for all predictor variables. However, the simultaneous models were not quite maximal in that, in order to avoid convergence failure, we did not include the correlation between the slope and the intercept. All analyses used the bobyqa optimizer, which also helped to reduce conversion failure. This strategy was successful in avoiding convergence failure, though several models yielded “singular fit” warnings, suggesting over-fitting. Nevertheless, in the interests of using an identical model each analysis, we did not simplify singular-fit models further. For example, for each language, the main analysis used the following lme4 syntax:

Rating ~ (1 + Preemption + Entrenchment + Semantics‖Participant) + (1|Verb) + Preemption + Entrenchment + Semantics.

Single-predictor models used the following syntax (for this example, the single-predictor evaluated is preemption):

Rating ~ (1 + Preemption‖Participant) + (1|Verb) + Preemption.

All three predictor variables were standardized into Z scores. The Semantics predictor referred to here is the main verb-semantics predictor (*event-merge*); the three additional semantics predictors (*autonomy*, *requires-causer*, and *directive*/*physical*) were assessed by means of exploratory (unregistered) analyses. The dependent variable for the main analysis was participants' raw sentence ratings on the 5-point judgment scale.

**Fig. 1:**
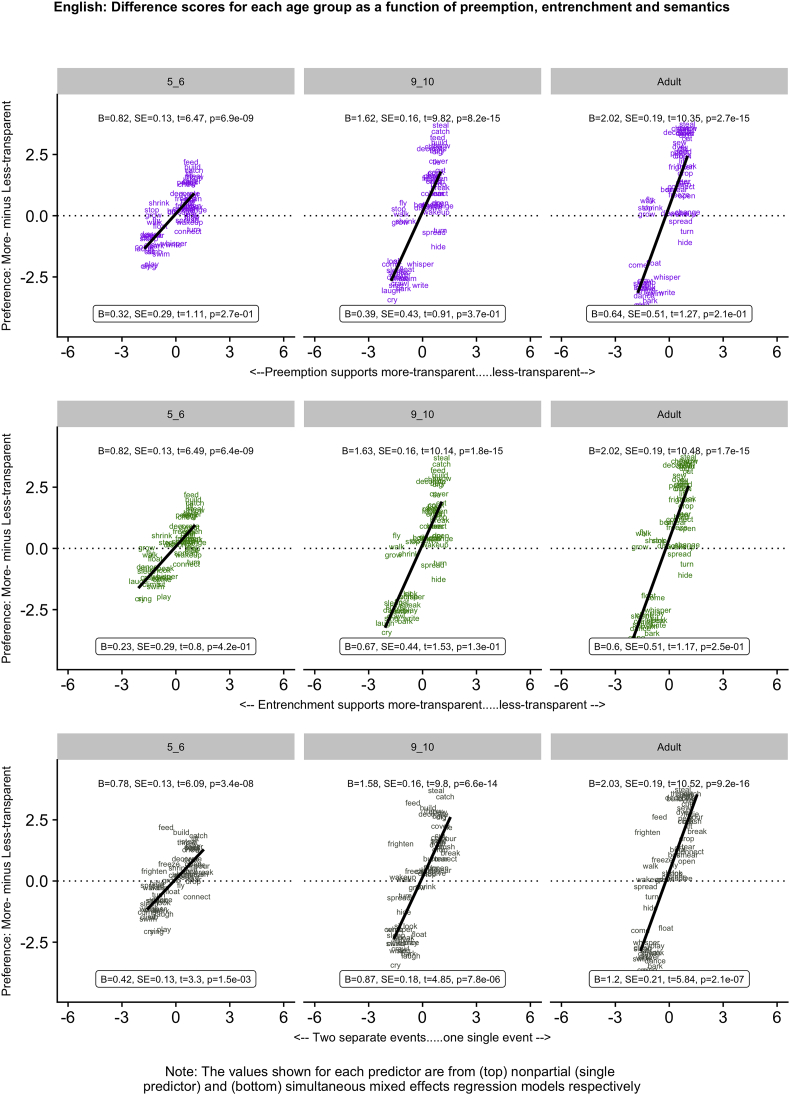
English: Difference scores for each age group as a function of preemption, entrenchment and semantics.

**Fig. 2:**
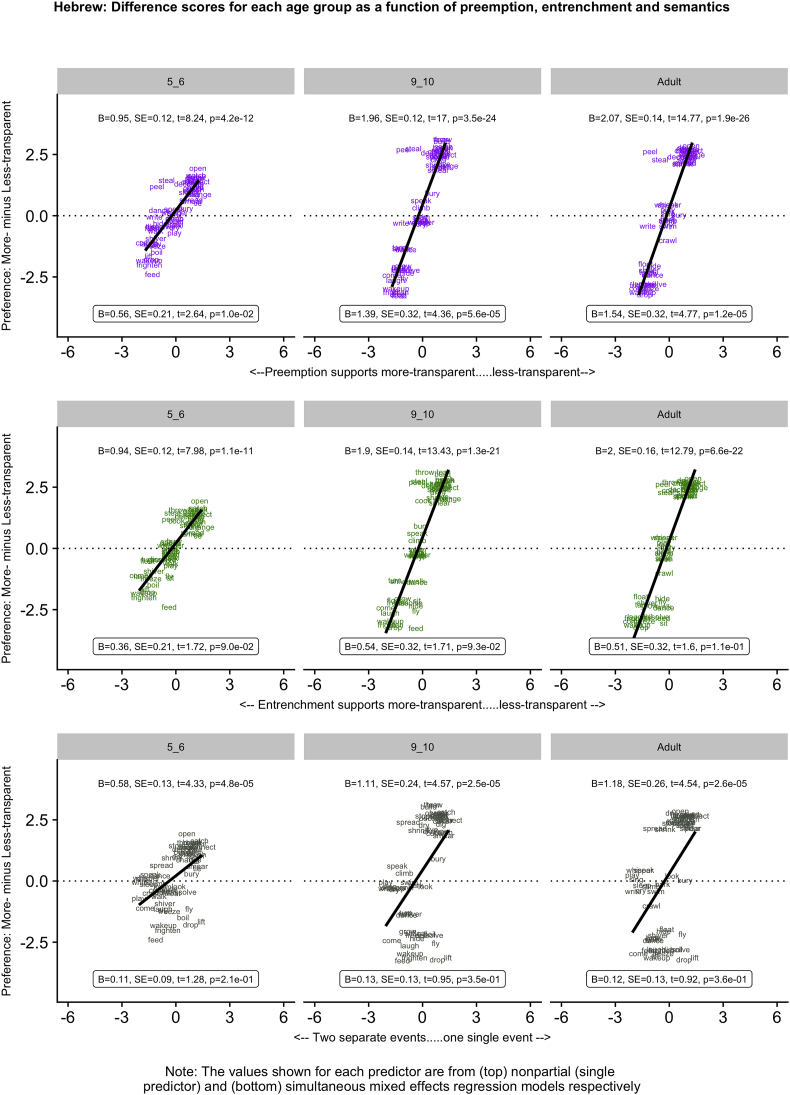
Hebrew: Difference scores for each age group as a function of preemption, entrenchment and semantics.

**Fig. 3:**
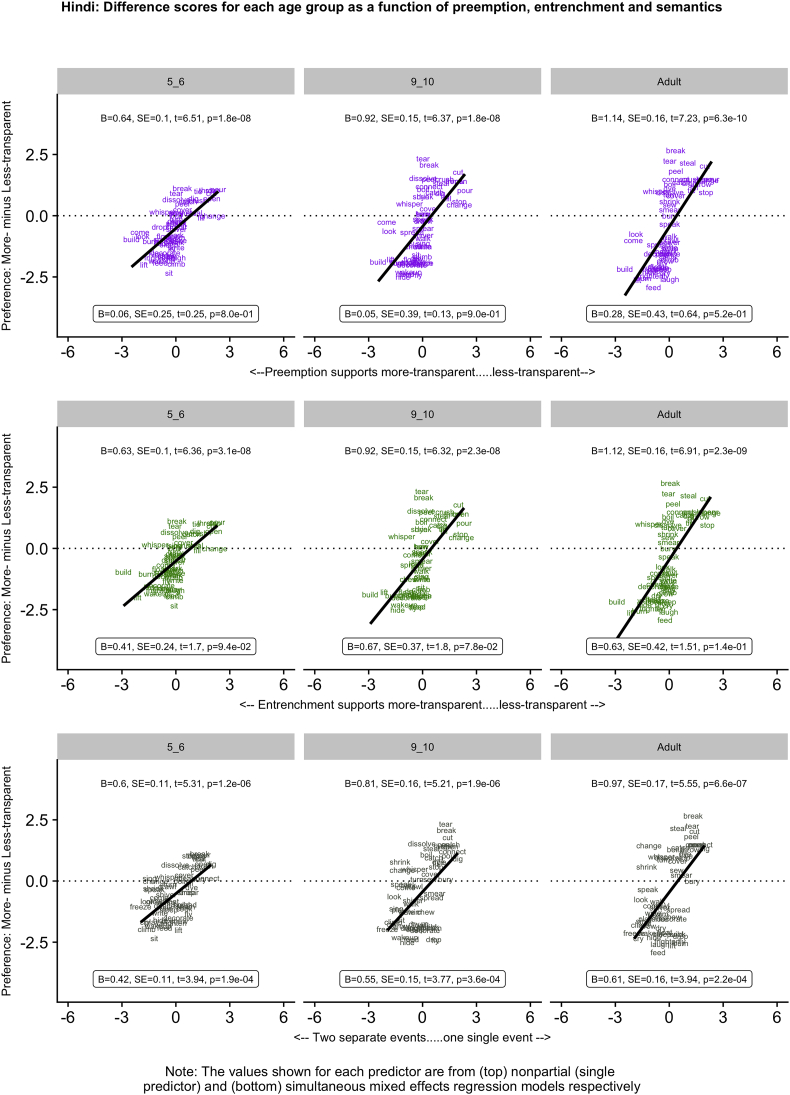
Hindi: Difference scores for each age group as a function of preemption, entrenchment and semantics.

**Fig. 4:**
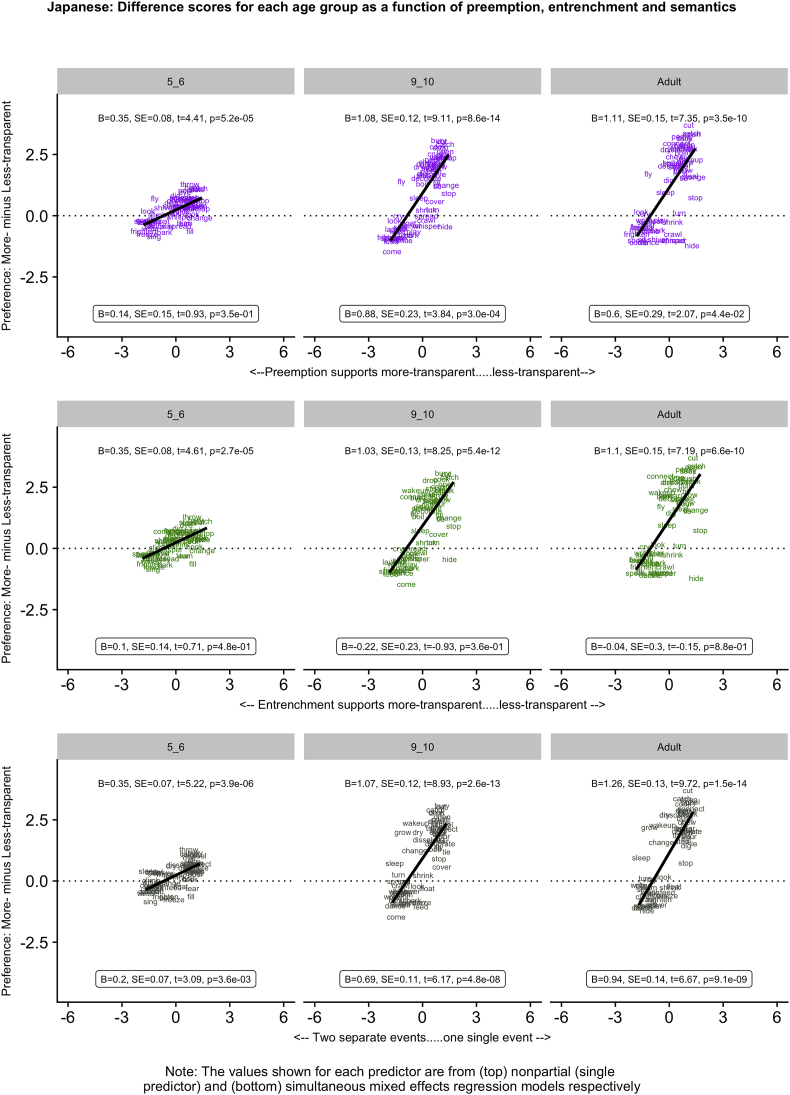
Japanese: Difference scores for each age group as a function of preemption, entrenchment and semantics.

**Fig. 5:**
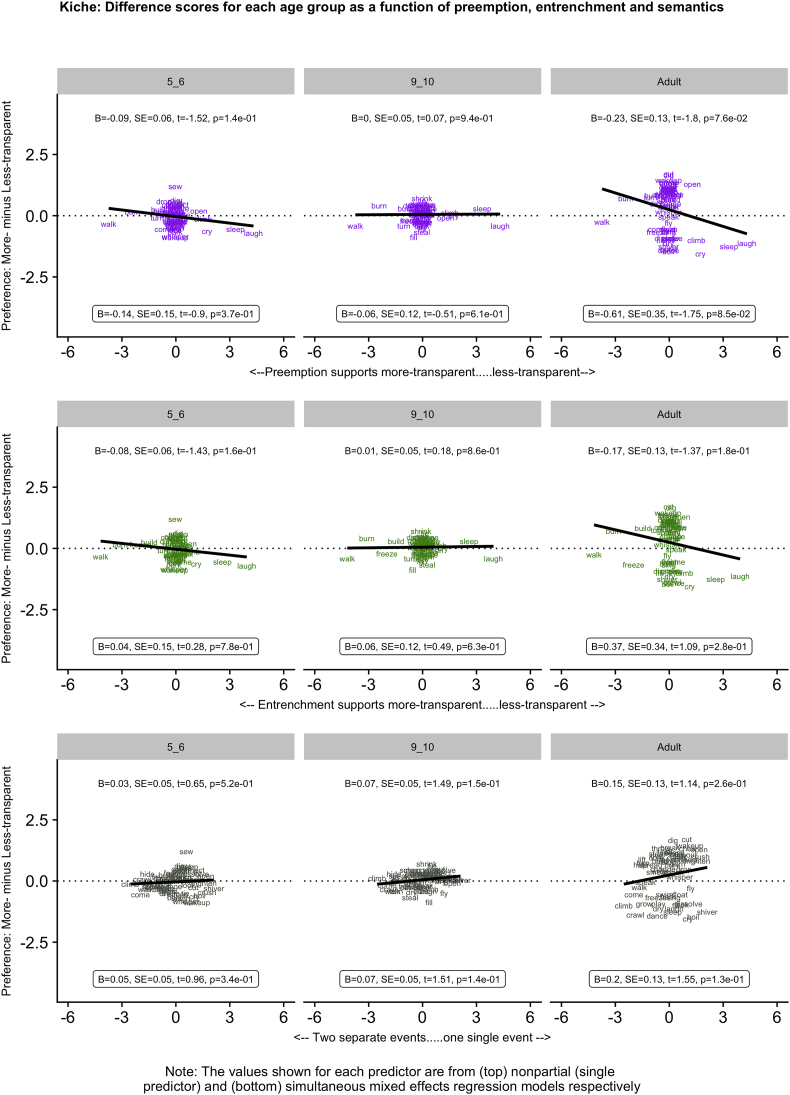
Kiche: Difference scores for each age group as a function of preemption, entrenchment and semantics.

Fig. 1, Fig. 2, Fig. 3, Fig. 4, Fig. 5 (English, Hebrew, Hindi, Japanese and K'iche') summarize the single-predictor (unboxed values, top) and simultaneous (boxed values, bottom) effects of preemption (purple), entrenchment (emerald) and event-merge semantics (ebony) in the difference-score analyses, which represent the easiest way to get a handle on the pattern of results. Single-predictor effects of the three supplementary semantic predictors, Autonomy (auburn), Directive (dark blue) and Requires (red) are presented in Appendix 3. The corresponding raw-score analyses, although designated the “main” analyses, are more detailed, and are therefore presented in Appendix 4 (less-transparent forms) and Appendix 5 (more-transparent forms) respectively. Correlations between predictors, which in some cases are indicative of significant collinearity, are presented in Appendix 6. The pattern of significant results is summarized in Table 4, and in the remainder of this section.

**Table 4:** Significant effects observed across languages and analyses.

Difference scores (see Fig. 1, Fig. 2, Fig. 3, Fig. 4, Fig. 5 for details)							
Language	Effect	Single-predictor model			Simultaneous model		
		5–6	9–10	Adults	5–6	9–10	Adults
English	Preemption	Y	Y	Y	N	N	N
	Entrenchment	Y	Y	Y	N	N	N
	Event-Merge	Y	Y	Y	Y	Y	Y
Hebrew	Preemption	Y	Y	Y	Y	Y	Y
	Entrenchment	Y	Y	Y	N	N	N
	Event-Merge	Y	Y	Y	N	N	N
Hindi	Preemption	Y	Y	Y	N	N	N
	Entrenchment	Y	Y	Y	N	N	N
	Event-Merge	Y	Y	Y	Y	Y	Y
Japanese	Preemption	Y	Y	Y	N	Y	Y
	Entrenchment	Y	Y	Y	N	N	N
	Event-Merge	Y	Y	Y	Y	Y	Y
K'iche'	Pre-emption	N	N	N	N	N	N
	Entrenchment	N	N	N	N	N	N
	Event-Merge	N	N	N	N	N	N


Supplementary semantic predictors (see Appendix 3 for details)				
Language	Effect	Single-predictor model		
		5–6	9–10	Adults
English	Autonomy of undergoer	Y	Y	Y
	Directive (verbal), not physical, causation	Y	Y	Y
	Requires external cause for event to occur	Y	Y	Y
Hebrew	Autonomy of undergoer	Y	Y	Y
	Directive (verbal), not physical, causation	Y	Y	Y
	Requires external cause for event to occur	Y	Y	Y
Hindi	Autonomy of undergoer	Y	Y	Y
	Directive (verbal), not physical, causation	Y	Y	Y
	Requires external cause for event to occur	Y	Y	Y
Japanese	Autonomy of undergoer	Y	Y	Y
	Directive (verbal), not physical, causation	Y	Y	Y
	Requires external cause for event to occur	Y	Y	Y
K'iche'	Autonomy of undergoer	Y	N	Y
	Directive (verbal), not physical, causation	Y	N	Y
	Requires external cause for event to occur	Y	N	Y


Less-transparent forms: main predictors (see Appendix 4 for details)							
Language	Effect	Single-predictor model			Simultaneous model		
		5–6	9–10	Adults	5–6	9–10	Adults
English	Preemption	Y	Y	Y	N	N	N
	Entrenchment	Y	Y	Y	Y	Y	Y
	Event-Merge	Y	Y	Y	N	Y	Y
Hebrew	Preemption	Y	Y	Y	N	N	N
	Entrenchment	Y	Y	Y	Y	Y	Y
	Event-Merge	Y	Y	Y	N	N	N
Hindi	Preemption	Y	Y	Y	Y	Y	Y
	Entrenchment	Y	Y	Y	N	N	N
	Event-Merge	Y	Y	Y	Y	Y	Y
Japanese	Preemption	Y	Y	Y	N	N	N
	Entrenchment	Y	Y	Y	N	Y	Y
	Event-Merge	Y	Y	Y	Y	Y	Y
K'iche'	Pre-emption	N	N	N	N	N	N
	Entrenchment	N	N	N	N	N	N
	Event-Merge	N	N	N	N	N	N


Less-transparent forms: supplementary semantic predictors (see Appendix 4 for details)				
Language	Effect	Single-predictor model		
		5–6	9–10	Adults
English	Autonomy of undergoer	Y	Y	Y
	Directive (verbal), not physical, causation	Y	Y	Y
	Requires external cause for event to occur	Y	Y	Y
Hebrew	Autonomy of undergoer	Y	Y	Y
	Directive (verbal), not physical, causation	Y	Y	Y
	Requires external cause for event to occur	Y	Y	Y
Hindi	Autonomy of undergoer	Y	Y	Y
	Directive (verbal), not physical, causation	Y	Y	Y
	Requires external cause for event to occur	Y	Y	Y
Japanese	Autonomy of undergoer	Y	Y	Y
	Directive (verbal), not physical, causation	Y	Y	Y
	Requires external cause for event to occur	Y	Y	Y
K'iche'	Autonomy of undergoer	N	N	Y
	Directive (verbal), not physical, causation	N	N	Y
	Requires external cause for event to occur	N	N	Y


More-transparent forms: main predictors (see Appendix 5 for details)							
Language	Effect	Single-predictor model			Simultaneous model		
		5–6	9–10	Adults	5–6	9–10	Adults
English	Preemption	Y	Y	Y	N	N	N
	Entrenchment	Y	Y	Y	N	Y	N
	Event-Merge	Y	Y	Y	N	Y	Y
Hebrew	Preemption	Y	Y	Y	Y	Y	Y
	Entrenchment	Y	Y	Y	N	N	N
	Event-Merge	N	N	N	N	Y	Y
Hindi	Preemption	Y	Y	Y	Y	N	N
	Entrenchment	N	Y	Y	N	Y	N
	Event-Merge	Y	Y	Y	Y	Y	N
Japanese	Preemption	Y	Y	Y	Y	N	N
	Entrenchment	N	Y	Y	N	N	N
	Event-Merge	Y	Y	Y	N	Y	Y
K'iche'	Pre-emption	Y	N	N	Y	N	Y
	Entrenchment	N	N	N	N	N	N
	Event-Merge	N	N	N	N	N	N


More-transparent forms: supplementary semantic predictors (see Appendix 5 for details)				
Language	Effect	Single-predictor model		
		5–6	9–10	Adults
English	Autonomy of undergoer	Y	Y	Y
	Directive (verbal), not physical, causation	Y	Y	Y
	Requires external cause for event to occur	Y	Y	Y
Hebrew	Autonomy of undergoer	N	N	N
	Directive (verbal), not physical, causation	N	N	N
	Requires external cause for event to occur	N	N	N
Hindi	Autonomy of undergoer	Y	N	N
	Directive (verbal), not physical, causation	N	N	N
	Requires external cause for event to occur	Y	Y	N
Japanese	Autonomy of undergoer	Y	Y	Y
	Directive (verbal), not physical, causation	Y	Y	Y
	Requires external cause for event to occur	Y	Y	Y
K'iche'	Autonomy of undergoer	Y	N	N
	Directive (verbal), not physical, causation	Y	N	N
	Requires external cause for event to occur	Y	N	Y

### 3.1 K'iche'

For K'iche', the only one of the three main predictors to show a significant effect was Preemption, and only for 5–6 year olds (single and simultaneous models) and adults (simultaneous model only), for more-transparent causative forms. The lack of Preemption and Entrenchment effects for K'iche' results largely from the fact that our corpus counts fail to capture enough instances of the target verbs (note in Fig. 5 how both predictors cluster tightly around zero). The null effect of Event-Merge Semantics is somewhat mystifying given that (a) K'iche' generally patterns as expected (and like the other languages) with regard to the three supplementary semantic predictors, each of which captures a subsidiary aspect of the notion of event-merge; and (b) as we will see shortly, Event-Merge ratings obtained from K'iche' speakers significantly predict difference scores for all other languages. Thus, our tentative conclusion is that, despite the null effect of Event-Merge, K'iche' does not, overall, seem to be radically different to the other four languages with regard to semantic effects.

### 3.2 Semantics

With the exception of K'iche', the main semantic predictor **Event-Merge** is almost always significant in single-predictor models (33/36 models), across languages, age-groups and forms (less-transparent/more-transparent/difference scores). The only exceptions (other than K'iche') are Hebrew less-transparent forms, for all age groups. In the main, this effect survives the more stringent simultaneous analysis (25/36 models), with the only exceptions (other than K'iche'), (a) less-transparent forms for Hebrew all age groups and English 5–6 year olds, (b) more-transparent forms for English, Hebrew and Japanese 5–6 year olds, and Hindi adults, and (c) difference scores for Hebrew (all age groups). That is, all but one of these null effects occurs for the youngest children and/or for Hebrew. The null effects for the youngest children are consistent with the possibility that they have yet to fully learn the relevant semantic properties of the verbs and/or the more- and less-transparent constructions, but also with the less interesting possibility that they simply show noisier performance on the judgment task.

The supplementary semantic predictors – (a) **Autonomy** of undergoer, (b) **Directive** (verbal), not physical, causation, and (c) **Requires** external cause for event to occur were significant for 42/45 difference-score models (these predictors are highly collinear with one another, and so were assessed in single-predictor models only). The null findings were all observed for K'iche' 9–10 year olds. The fact that both the younger and older K'iche' speakers nevertheless showed the predicted effects suggests that the null finding for the 9–10 year olds was a blip (perhaps related to the particular children studied), particularly given that none of the other languages showed any suggestion of U-shaped development. The by-sentence-type analyses showed that the significant effects observed resulted mainly from effects of the supplementary semantic predictors on less-transparent causative forms (39/45 significant effects, with the six null effects all for the two youngest K'iche' groups), rather than more-transparent causative forms (25/45). Broadly speaking, English and Japanese speakers showed these effects, while Hebrew, Hindi (except for Requires) and K'iche' (except for 5–6 year olds) did not. This pattern probably reflects the fact that more-transparent casuativization is less “choosy” than less-transparent causativization with regard to the verbs to which it applies.

### 3.3 Preemption and entrenchment

With the exception of K'iche', both of these predictors are almost always significant in single-predictor models (70/72 models), across languages, age-groups and forms (less-transparent/more-transparent/difference scores). The only exceptions (other than K'iche') are entrenchment for more-transparent forms for Hindi and Japanese 5–6 year olds. Presumably this simply reflects noisy performance by the youngest children, since all groups of 5–6 year olds (except K'iche') showed entrenchment effects for both more-transparent forms and difference scores. In the main, these effects did not survive the more stringent simultaneous analyses (just 23/72 analyses). This is no doubt because the preemption and entrenchment predictors are extremely highly correlated (see Appendix 6); so much so, that we can have little faith in the ability of the simultaneous models to separate them. As demonstrated by Westfall and Yarkoni (2016), when predictors are highly correlated, they can be separated using regression techniques only when they are measured essentially *perfectly*. In the present study, both Preemption and Entrenchment are measured extremely *imperfectly*, because both are estimated on the basis of corpus data, rather than the input language to which the participants were actually exposed. As one would expect then, the simultaneous-model data suggest no clear winner: Preemption is significant in 13 models, Entrenchment in 10 (never both in the same model) with no clear pattern in terms of languages, age-groups or forms.

However, it is important to emphasize that this does not mean that the preemption and entrenchment hypotheses are not supported by the present dataset. What the comparison between the single-predictor and simultaneous models is telling us – together with the predictor-correlation data shown in Appendix 6 – is that both preemption and entrenchment are significant predictors of participants' judgments, but cancel each other out in the simultaneous models. That is, because they largely explain the same variance in participants' judgments, both preemption and entrenchment are robbed of the opportunity to account for variance accounted for by the other. To confirm this intuition, we ran a series of unplanned, non-registered analyses (on the difference scores only) with only one of the two statistical predictors – either preemption or entrenchment – retained in the simultaneous model (alongside Event Merge semantics). These analyses (see Appendix 7) confirmed that, with the exception of K'iche', preemption is always significant (*p* < 0.01) in a two-predictor model with entrenchment removed and vice versa. Indeed, even for K'iche', preemption narrowly reached significance for adults (*p* = 0.047). Thus, the appropriate conclusion is that, except for K'iche', preemption or entrenchment is always operational (in addition to semantics) but that, due to collinearity between these predictors, we cannot tell which. The relationship between the two is however clarified considerably by the computational modeling work presented below.

### 3.4 Supplementary (unplanned) analysis: all-languages analysis

Next, in an unplanned, non-preregistered analysis, we collapsed all languages except for K'iche' (for which we already know no predictors are significant) into a single analysis, including language, and its interactions with Preemption, Entrenchment and Event-Merge, as fixed effects. Because it is already clear that, with very few exceptions, all three predictors are always significant in single-predictor models, this analysis used simultaneous models only. These models are summarized in Table 5.

**Table 5:** All-language analysis.

Difference scores	Age 5–6		df	t	p	Age 9–10		df	t	p	Adults		df	t	p
	Est	SE				Est	SE				Est	SE			
Intercept	0.08	0.07	179	1.21	0.23	0.16	0.09	144	1.84	0.07	0.39	0.09	79	4.24	**0.00**
Preemption	0.35	0.18	1317	1.90	0.06	0.57	0.16	2220	3.63	**0.00**	0.82	0.15	2139	5.49	**0.00**
Lang = Hebrew	0.15	0.08	185	1.83	0.07	0.30	0.09	184	3.43	**0.00**	−0.06	0.07	184	−0.86	0.39
Lang = Hindi	−0.60	0.08	189	−7.54	**0.00**	−0.60	0.09	187	−6.82	**0.00**	−0.85	0.07	187	−12.48	**0.00**
Lang = Japanese	0.15	0.08	185	1.84	0.07	0.77	0.09	185	8.75	**0.00**	0.77	0.07	184	11.28	**0.00**
Entrenchment	0.24	0.18	1315	1.39	0.16	0.47	0.15	2344	3.06	**0.00**	0.32	0.14	2301	2.25	**0.02**
Event-Merge	0.36	0.08	345	4.46	**0.00**	0.73	0.08	488	9.64	**0.00**	0.94	0.07	602	13.32	**0.00**
Pre*Hebrew	0.16	0.24	1099	0.68	0.50	0.75	0.21	1851	3.64	**0.00**	0.69	0.20	1756	3.53	**0.00**
Pre*Hindi	−0.22	0.23	1206	−0.94	0.35	−0.22	0.20	1557	−1.14	0.25	−0.18	0.18	1413	−0.96	0.34
Pre*Japanese	−0.05	0.23	1133	−0.22	0.83	0.42	0.19	1554	2.19	**0.03**	−0.09	0.18	1411	−0.49	0.63
Ent*Hebrew	0.18	0.23	1093	0.79	0.43	0.14	0.20	1936	0.69	0.49	0.18	0.19	1863	0.95	0.34
Ent*Hindi	0.11	0.22	1159	0.49	0.63	−0.04	0.18	1586	−0.23	0.82	0.03	0.17	1452	0.17	0.87
Ent*Japanese	−0.36	0.22	1144	−1.66	0.10	−0.79	0.19	1690	−4.24	**0.00**	−0.57	0.18	1574	−3.22	**0.00**
E-M*Hebrew	−0.28	0.10	269	−2.69	**0.01**	−0.74	0.09	288	−8.41	**0.00**	−1.09	0.08	281	−14.05	**0.00**
E-M*Hindi	0.03	0.10	275	0.30	0.76	−0.34	0.09	303	−3.83	**0.00**	−0.64	0.08	295	−8.15	**0.00**
E-M*Japanese	−0.14	0.10	296	−1.37	0.17	−0.21	0.09	316	−2.33	**0.02**	−0.30	0.08	311	−3.75	**0.00**

Less-transparent															
Intercept	3.47	0.08	221	42.32	**0.00**	3.71	0.06	190	60.27	**0.00**	3.70	0.07	150	55.14	**0.00**
Preemption	−0.06	0.11	1437	−0.60	0.55	−0.12	0.08	1636	−1.43	0.15	−0.14	0.07	1805	−1.93	0.05
Lang = Hebrew	−0.04	0.11	187	−0.35	0.73	−0.44	0.07	186	−6.27	**0.00**	−0.38	0.07	183	−5.47	**0.00**
Lang = Hindi	−0.27	0.11	188	−2.58	**0.01**	−0.14	0.07	187	−2.06	**0.04**	−0.26	0.07	184	−3.73	**0.00**
Lang = Japanese	0.25	0.11	186	2.35	**0.02**	0.46	0.07	186	6.62	**0.00**	0.69	0.07	183	9.88	**0.00**
Entrenchment	0.59	0.13	1634	4.70	**0.00**	0.99	0.09	2087	10.90	**0.00**	1.14	0.08	2290	13.63	**0.00**
Event-Merge	0.06	0.07	433	0.86	0.39	0.37	0.05	590	7.26	**0.00**	0.32	0.05	664	6.89	**0.00**
Pre*Hebrew	0.01	0.13	1137	0.11	0.91	0.05	0.10	1267	0.52	0.60	0.09	0.09	1394	0.99	0.32
Pre*Hindi	0.47	0.14	1243	3.36	**0.00**	0.75	0.11	1431	7.06	**0.00**	0.87	0.10	1585	8.89	**0.00**
Pre*Japanese	0.20	0.12	812	1.66	0.10	0.33	0.09	876	3.57	**0.00**	0.23	0.09	996	2.65	**0.01**
Ent*Hebrew	0.22	0.17	1389	1.29	0.20	0.55	0.12	1647	4.60	**0.00**	0.40	0.11	1817	3.64	**0.00**
Ent*Hindi	−0.78	0.16	1162	−4.91	**0.00**	−1.20	0.11	1383	−10.62	**0.00**	−1.28	0.10	1535	−12.44	**0.00**
Ent*Japanese	−0.70	0.15	863	−4.81	**0.00**	−0.76	0.10	1032	−7.46	**0.00**	−0.98	0.09	1160	−10.51	**0.00**
E-M*Hebrew	−0.14	0.09	446	−1.57	0.12	−0.51	0.07	550	−7.56	**0.00**	−0.47	0.06	523	−7.78	**0.00**
E-M*Hindi	0.19	0.08	292	2.30	**0.02**	−0.01	0.06	358	−0.19	0.85	0.03	0.05	342	0.49	0.63
E-M*Japanese	0.10	0.08	297	1.30	0.19	−0.13	0.06	365	−2.19	**0.03**	−0.12	0.05	347	−2.34	**0.02**

More-transparent															
Intercept	3.39	0.09	227	35.92	**0.00**	3.56	0.09	229	40.21	**0.00**	3.32	0.08	204	40.95	**0.00**
Preemption	0.21	0.07	1096	2.92	**0.00**	0.28	0.06	1058	4.38	**0.00**	0.40	0.06	976	6.36	**0.00**
Lang = Hebrew	−0.18	0.12	187	−1.48	0.14	−0.74	0.11	187	−6.94	**0.00**	−0.32	0.09	185	−3.57	**0.00**
Lang = Hindi	0.33	0.12	188	2.70	**0.01**	0.46	0.11	188	4.25	**0.00**	0.59	0.09	187	6.54	**0.00**
Lang = Japanese	0.09	0.12	187	0.77	0.44	−0.30	0.11	187	−2.84	**0.00**	−0.08	0.09	185	−0.88	0.38
Entrenchment	0.00	0.06	594	−0.02	0.98	0.11	0.05	758	2.29	**0.02**	0.01	0.04	4955	0.30	0.76
Event-Merge	−0.17	0.06	549	−2.80	**0.01**	−0.26	0.06	503	−4.42	**0.00**	−0.48	0.06	516	−8.45	**0.00**
Pre*Hebrew	0.12	0.09	942	1.33	0.18	0.55	0.08	816	6.70	**0.00**	0.53	0.08	744	6.54	**0.00**
Pre*Hindi	0.04	0.09	701	0.45	0.65	−0.05	0.08	626	−0.64	0.52	−0.10	0.08	579	−1.34	0.18
Pre*Japanese	−0.01	0.09	755	−0.14	0.89	0.02	0.08	639	0.20	0.84	−0.09	0.08	589	−1.21	0.23
Ent*Hebrew	0.15	0.08	548	1.82	0.07	0.10	0.06	691	1.63	0.10	0.18	0.06	5331	2.98	**0.00**
Ent*Hindi	−0.01	0.07	400	−0.10	0.92	0.04	0.06	463	0.75	0.45	0.15	0.05	5388	2.80	**0.01**
Ent*Japanese	−0.07	0.08	442	−0.87	0.39	0.06	0.06	498	1.03	0.30	0.15	0.06	5334	2.63	**0.01**
E-M*Hebrew	0.27	0.08	510	3.56	**0.00**	0.63	0.07	356	8.60	**0.00**	0.95	0.07	344	13.53	**0.00**
E-M*Hindi	0.01	0.07	432	0.08	0.93	0.08	0.07	315	1.20	0.23	0.35	0.07	305	5.18	**0.00**
E-M*Japanese	0.12	0.07	405	1.63	0.10	0.01	0.07	296	0.13	0.90	0.01	0.07	287	0.15	0.88

Effects shown in bold are signifant at p<0.05 or better.

In terms of main effects, the findings generally echo the individual by-language analyses, yielding – in most cases – an effect of Event-Merge and either Preemption or Entrenchment, though usually not both (due to collinearity). In terms of interactions, focussing on difference scores – which is a good way to neutralize differences in absolute ratings for corresponding forms across different languages – Preemption, Entrenchment and Event-Merge effects were compared to English, the arbitrarily chosen reference category. As compared to English, Preemption effects were larger for Hebrew (9–10-year-olds and adults) and Japanese (9–10-year-olds only). Entrenchment effects were smaller in Japanese (9–10-year-olds and adults). Interestingly, for both 9–10-year-olds and adults, Event-Merge effects were smaller for all three languages – Hebrew, Hindi and Japanese – than for English (and likewise for Hebrew 5–6 year olds). It would be unwise to draw strong theoretical claims on the basis of these unplanned analyses, for which no specific predictions were made. However, it would seem safe to say that, although effects of Preemption, Entrenchment and Semantics are generally observed across languages, different languages are free to draw different boundaries with exactly how much flexibility is allowed in the system.

In this regard, it is interesting to note that – as indicated by the fixed effects for language – Japanese and Hindi (at least for the two older groups) have respectively larger and smaller difference scores across the board than English. Of course, we cannot rule out the possibility that these different populations simply approach the rating task differently. If taken at face value, however, these findings suggest that Japanese is more rigid than English, generally allowing only the more-transparent or only the less-transparent causative form of each verb root. Conversely, Hindi is more flexible than English, often allowing both the more- and less-transparent causative form of each verb root. Hebrew, at least on the basis of the adult ratings, sits alongside English in between Japanese and Hindi.

### 3.5 Supplementary (unplanned) analysis: Event-Merge as a crosslinguistic semantic universal

As a second unplanned analysis, we decided to investigate whether, as proposed by Shibatani and Pardeshi (2002), Event-Merge operates as a semantic constraint across languages, we investigated the ability of Event-Merge ratings collected for each language to predict the grammaticality judgments (adult difference scores) for each of the other languages. Since this is only an exploratory analysis, we conducted correlations on the mean scores for each verb, rather than running a full suite of 25 further mixed-effects models. The results are summarized in Table 6.

**Table 6:** Crosslinguistic event-merge analysis.

	English Diff	Hebrew Diff	Hindi Diff	Japanese Diff	K'iche' Diff
English Event-Merge	0.82	0.79	0.71	0.83	0.19
Hebrew Event-Merge	0.60	0.52	0.57	0.55	0.14
Hindi Event-Merge	0.57	0.50	0.60	0.49	0.21
Japanese Event-Merge	0.85	0.79	0.70	0.81	0.34
K'iche' Event-Merge	0.54	0.54	0.61	0.58	0.15

Note: Critical r (df = 58) value for *p* < 0.05 = 0.21; for *p* < 0.01 = 0.30 (one tailed).

This analysis revealed that, in every case, event-merge ratings obtained from speakers of one language significantly predicted adult difference scores obtained across all four other languages. Furthermore, the intra-language correlations were, if anything, generally slightly larger than the inter-language correlations. Indeed, the only correlation not to reach significance was between K'iche' Event-Merge ratings and K'iche' difference scores, even though K'iche' Event-Merge ratings were correlated with difference scores from all four other languages at *p* < 0.01. Although the K'iche' findings are difficult to explain, overall, this analysis constitutes evidence for Shibatani and Pardeshi's (2002) claim that, with regard to causativity, Event-Merge constitutes something approaching a semantic universal (though, of course, a wider sample of unrelated languages^6^ would be needed to test this claim more fully).

Before moving on, it is important to acknowledge three potential objections to the operationalization of the predictors in the present study. First, we conceptualized preemption rather narrowly as involving only two forms of the same root (e.g., *break* and *make break* for English; *toR* and *Tutaa* for Hindi). In real life, overgeneralized forms (e.g., *She died him) are also preempted by forms with different roots (e.g., *She killed him*). It was not possible within the time constraints of the study to generate and count all such possible alternative competitors. Rather, we sought to minimize the problem by selecting only verbs that can potentially appear in more- and less-transparent forms with the same root in all languages (i.e., we avoided pairs like *kill*/*die*).

Second, for both the preemption and entrenchment predictors, the base rates (shown in Table 3, Table 4 as “all other verbs”), are calculated not for all other verbs in the language as a whole, but only all other verbs in our verb set. Although the former would have been preferable, such counts are impossible to obtain using automated procedures. We attempted to mitigate against this problem by selecting verbs that are, for each language, split relatively evenly between more-transparent-preferring, less-transparent-preferring and ambivalent, and thus as representative as possible of verbs more generally. This operationalization also blurs somewhat the distinction between preemption and entrenchment as the latter predictor incorporates not only the overall frequency of the verb but the notion of expected frequency in a particular target construction. That said, as we argue in the General Discussion, it may indeed be appropriate to see preemption and entrenchment more as two sides of the same coin.

Third, it is difficult to be sure exactly what was driving participants' behaviour in the semantic rating tasks. As noted by an anonymous reviewer, the very act of providing a rating may cause participants to focus on particular properties of an event that are not always salient in more naturalistic contexts. Furthermore, although we maintain that it was important not to show the causer, so as to avoid biasing participants' responses, we acknowledge that leaving the causer up to the participants' imaginations is likely to have led to some idiosyncratic ratings. That said, the finding that these semantic ratings were able to predict participants' grammaticality judgments even across languages confirms that whatever semantics these ratings were capturing – and however indirectly – they were indeed (morpho-)syntactically relevant.

Finally, it is worth considering the extent to which these results have implications for (a weak version of) the famous Sapir-Whorf hypotheses that language shapes thought. Do speakers of a particular language view a particular pair of causing + caused events as merged *because* that language merges the two with no need for an overt causative morpheme. Or, vice-versa, do languages merge a particular pair of causing + caused events into a less-transparent causative because humans, in general, conceptualize them as one? As a cross-sectional, correlational study, the present findings speak only indirectly to this question. On our view, however, they are more consistent with the second “meaning-first” view, given the finding that the Event-Merge ratings from each language predict the grammaticality judgments scores from the others. Consistent with this view, note that Japanese appears to be in the middle of a lexicalization process, visible in the fact that most lexical (less-transparent) causatives contain some reduced form of the causative marker *-*(*s*)*ase*. This suggests that, over time, verbs with Event-Merge semantics lose overt causative marking rather than the Sapir-Whorf alternative (i.e., verbs arbitrarily losing overt causative marking, causing an increase in perceived Event-Merge semantics).

### 3.6 Summary of judgment results

In summary, the present data suggest that any successful crosslinguistic account of how children learn to mark causativity while (eventually) avoiding overgeneralization errors must yield effects of verb Semantics (Event-Merge and/or its subsidiary predictors showed significant effects within and across languages) and of Preemption and/or Entrenchment (significant for all languages, though only sporadically for K'iche'), although – due to collinearity between the predictors – we cannot tell which. The aim of work described below was therefore to develop such an account, instantiated as a computational model.

### 3.7 Computational modeling

Our starting point when seeking to develop a computational model was the discriminative learning framework outlined in, for example, Ramscar and Yarlett (2007). One advantage of this framework is that it has already been used to model a number of important phenomena in the child language acquisition literature, including grammatical gender (Arnon & Ramscar, 2012), word-learning (e.g., Baayen, Chuang, Shafaei-Bajestan, & Blevins, 2019; Ramscar, Dye, & Klein, 2013), and both inflectional and derivational morphology (e.g., Baayen & Smolka, 2019; Milin, Divjak, Dimitrijević, & Baayen, 2016; Ramscar, Dye, & McCauley, 2013; Ramscar & Yarlett, 2007). A second advantage of the framework is its simplicity. Unlike, for example, three-layer connectionist networks (e.g., Engelmann et al., 2019), discriminative learning models do not incorporate hidden units. Indeed, for the present simulations, we found that adding a hidden layer made virtually no difference to the results. It is important to note, however, that such a simple model is possible only because we conceptualize the task at a rather high level: The model's tasks is simply to learn, for each verb, which of two pre-given causative forms is preferred. Children, of course, face the additional task of learning the forms themselves. Nevertheless, the use of a high-level task is important because it allows us to use an identical model architecture for all languages. A third advantage of this framework is that it is well grounded in the domains of both human and animal learning generally (e.g., Rescorla & Wagner, 1972; Rescorla, 1988; Gureckis & Love, 2010), and so enjoys psychological plausibility as a model of learning (unlike, for example, models that conceptualize the problem in terms of high-level Bayesian inference; e.g., Hsu & Chater, 2010). One departure from most discriminative learning models is that semantic features are represented not with cues that are either present or absent (i.e., 1/0), but by four units with continuous activation (i.e., using a form of the Widrow-Hoff, rather than Rescorla-Wagner, learning rule).

**Fig. 6:**
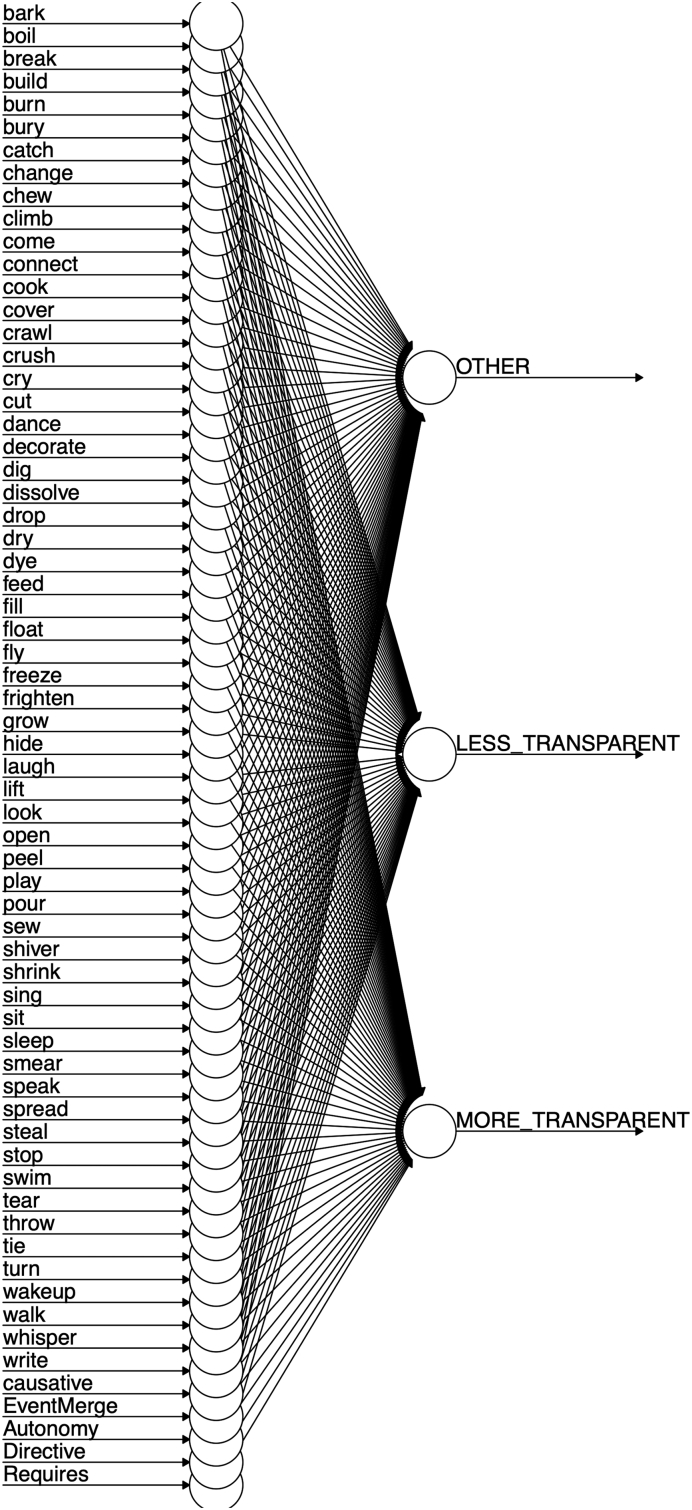
Architecture of the computational model.

It is important to stress that the model is never presented with participants' grammaticality judgment data (which would make the learning task trivial, adding nothing beyond the linear regression models already reported). Rather, each input-output pair presented to the model represents an utterance in the corpus used for each language (see Methods above). The architecture of the model, which is identical for each language, is shown in Fig. 6. The input to the model is a vector of 60 lexical units (1/0), a causative unit (1/0) and four semantic units (continuous activation level 0–1). The orthogonal lexical units represent the identity of the verb, and can be conceptualized as a pseudo-phonological representation (e.g. the root /bɔɪl/ for English *boil*) and/or a pseudo-lexical-semantic representation (e.g., “[of a liquid] to heat until it reaches boiling point”). The causative unit (1/0) indicates whether or not the utterance presented to the model on that trial conveys causation. That is, the causative unit is set to 1 if the corpus utterance uses either the more- or less-transparent causative form for the relevant verb (e.g., *The man made the water boil; The man boiled the water*) and to 0 if it does not (e.g., *The water boiled*). This unit can be conceptualized as representing, at a very broad-brush level, event-level semantics. The four semantic units, Event-Merge, Autonomy, Directive and Requires, are assigned a continuous activation level based on the mean rating – across all semantic raters for the relevant language – for the verb in the relevant utterance. These units can be conceptualized as representing some subset of the overall semantics (i.e., verb-level and/or event-level semantics) of the relevant utterance. Finally, the orthogonal output units (with softmax activation function) are each set to 1 or 0 representing the form/utterance type of the relevant corpus utterance: More transparent (e.g., *The man made the water boil*), Less transparent (*The man boiled the water*) or Other (e.g., *The water boiled*).

Conceptually speaking, the model instantiates preemption in terms of the competition between the More- and Less-transparent output units when the Causative input unit is set to 1. For example, the input utterance *The man boiled the water* strengthens the mapping of boil + causative → Less transparent at the expense of boil + causative → More transparent. The model instantiates entrenchment in terms of the competition between the Other output unit and the More- and Less-transparent output units when the Causative input unit is set to 0. For example, the input utterance *The water boiled* strengthens the mapping of boil → Other at the expense of both boil → More-transparent and boil → Less-transparent. The model instantiates verb semantics in that higher scores on EventMerge, Autonomy, Directive and Requires are (if Shibatani & Pardeshi's, 2002, characterization is correct) predictive of less- rather than more-transparent causation.

For each language, we ran a total of 4800 models: 2400 investigating the model's ability to learn the full training set of 60 verbs, 2400 investigating its ability to generalize to unseen verbs in a split-half validation test (i.e., training on one randomly-selected set of 30 verbs, testing on the 30 held-out verbs). In each case, the 2400 models constituted 48 models (representing our 48 human participants per age group) for each of 50 epochs (representing different stages of development). Each epoch consisted of 10,000 input “utterances” randomly selected from the relevant corpus (with-replacement selection was used, since some of the corpora were small), presented in random order. Thus, the computational models – like the statistical models presented earlier – do not receive information about verbs in the language other than the 60 trained (since the necessary corpus counts cannot be obtained automatically). Models were implemented using the *nnet* R package (Venables & Ripley, 2013) with both range and decay parameters set to 0.5.

It is important to stress again that the model was not provided with any grammaticality judgment data; its task was simply to learn which verbs and semantic features predict which output forms (More-Transparent, Less-Transparent, Other), for causative and non-causative scenes/events. At test, the model was, in effect, asked to rate the relative acceptability of the More-transparent causative, Less-transparent causative and Other form/sentence of each verb, as a description of a causative event/scene. That is, the model – with learning switched off – was presented in turn with each verb (i.e., the relevant combination of lexical units and verb semantic units) with the Causative unit set to 1, and the resulting activation levels of the More-transparent, Less-Transparent and Other output units taken as its acceptability judgment for the relevant sentence (e.g., *Someone made the water boil; Someone boiled the water*; *The water boiled*). Model performance was assessed by correlating these ratings with human judgment data, taking the mean across all 48 participants in each age group.

## 4 Results and discussion (computational modeling)

**Fig. 7:**
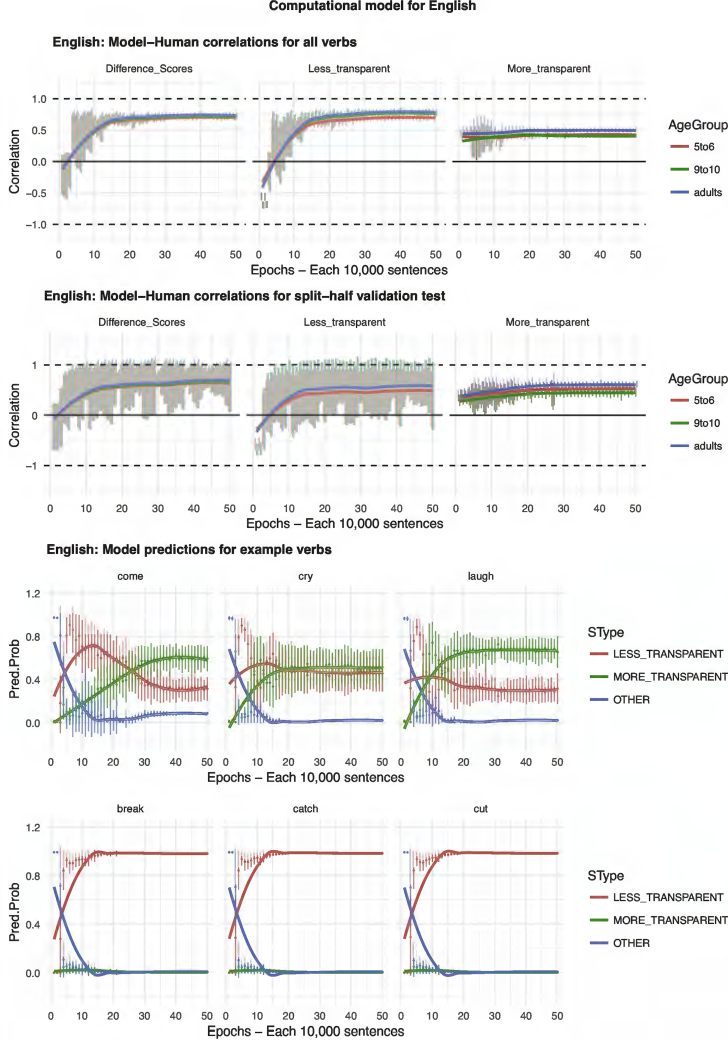
Computational model for English.

**Fig. 8:**
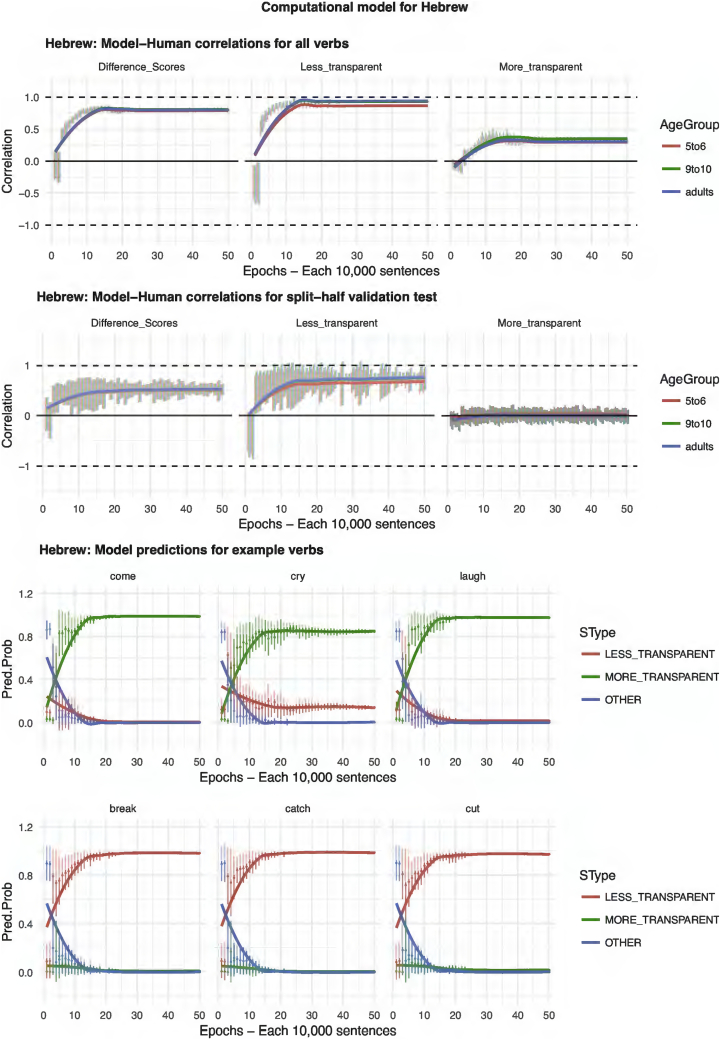
Computational model for Hebrew.

**Fig. 9:**
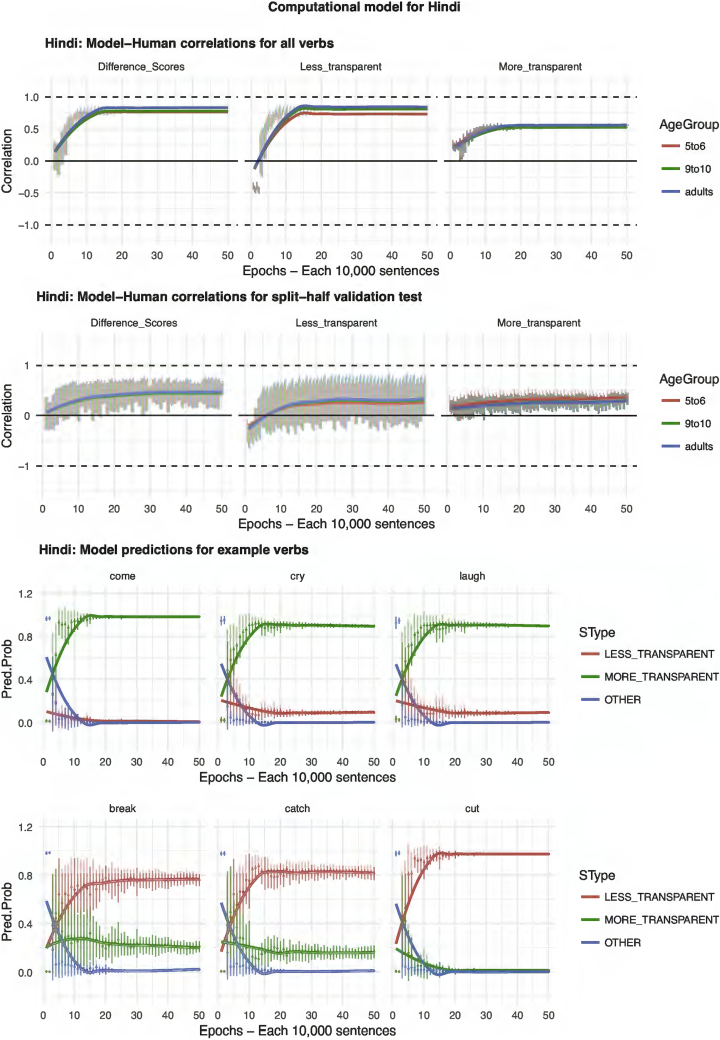
Computational model for Hindi.

**Fig. 10:**
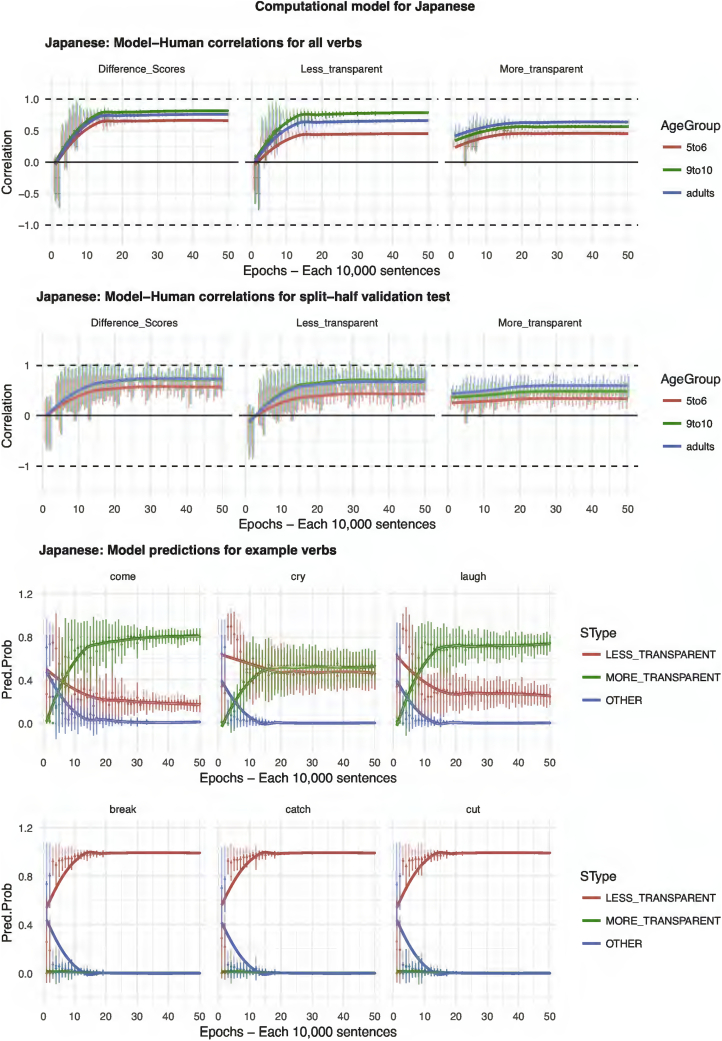
Computational model for Japanese.

**Fig. 11:**
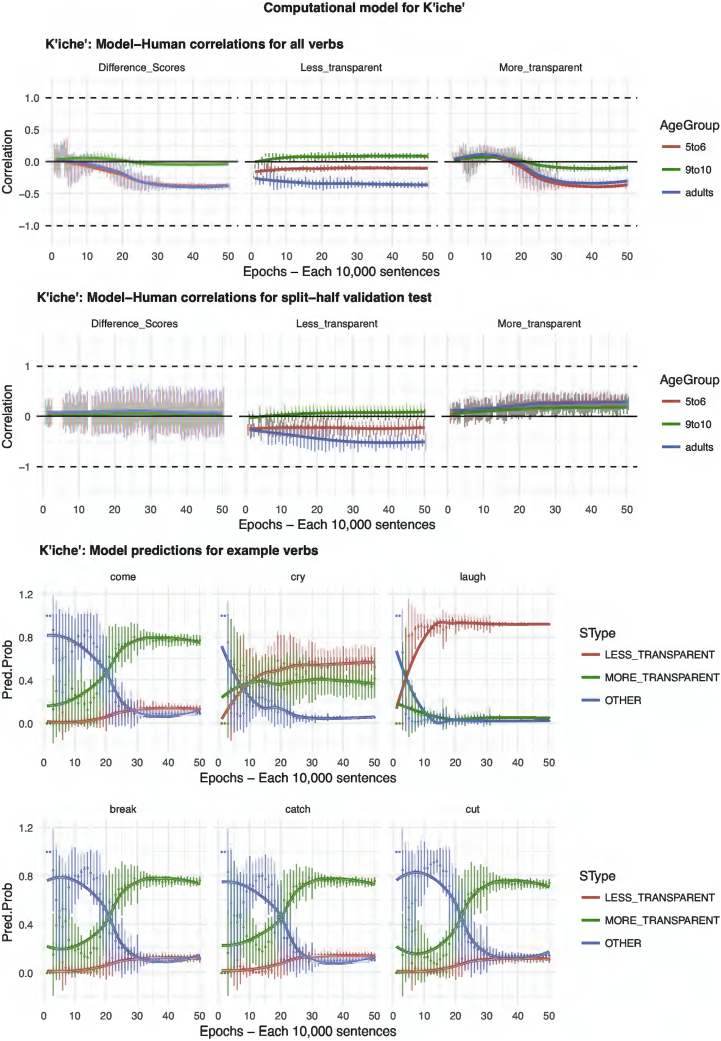
Computational model for K'iche'.

The results of the computational modeling are summarized in Fig. 7, Fig. 8, Fig. 9, Fig. 10, Fig. 11. Each figure shows performance on (top) the full set of 60 verbs (middle) the split-half validation test (30 verbs) and (bottom) verb-by-verb performance for six verbs chosen to exemplify, across languages, verbs that generally prefer more-transparent (come, cry, laugh) and less-transparent (break, catch, cut) causative. For K'iche', the model performs poorly, showing virtually zero correlation with human judgments (though recall from the analysis of human judgment data that, for K'iche' the corpus counts are highly unreliable, and the Event-Merge ratings do not predict acceptability judgments). For English, Hebrew, Hindi and Japanese, the models show excellent – and remarkably similar – performance. Looking at difference scores (in many ways the fairest test of the model, which really makes relative rather than absolute predictions regarding the acceptability of the more- and less-transparent causative forms), all four models achieve correlations with human performance of around *r* = 0.75 or better when tested on all 60 verbs. Even when tested on verbs it has never seen, in the split-half validation test, all models (except, again, K'iche') show correlations of around *r* = 0.50 with human performance in the difference-score analysis. Turning to raw scores, the models' predictions for less-transparent causative forms are generally on a par with its predictions for difference scores. It is only for more-transparent causative forms that performance dips somewhat, though correlations remain in the region of *r* = 0.3–0.5.

Zooming in on the six example verbs (bottom panels of Fig. 7, Fig. 8, Fig. 9, Fig. 10, Fig. 11) reveals how the model achieves its performance. Following a very brief early period in which its best guess is an Other (non-causative) form (presumably because such forms are considerably more frequent in the corpus than either causative form), the model learns that, when the Causative unit is set to 1 (as is always the case on test trials), either a more- or less-transparent causative form is required. Interestingly, for verbs that require a less-transparent causative form (*come*, *cry* and *laugh*), the English, Hebrew and Japanese models show a brief period of overgeneralization, analogous to errors such as *Someone came/*cried*/*laughed the boy* reported for children (at least for English; e.g., Bowerman, 1988). Hindi does not show this period but, at least for *cry* and *laugh*, does pass through a short period in which the two forms have roughly equal activation. On the other hand, no model shows a comparable overgeneralization period for verbs that require a more-transparent causative form (*break*, *catch*, *cut*). Accordingly, we are not aware of any such errors (e.g. *?Someone made the vase break*; *Someone made the ball catch; *Someone made the paper cut) produced by children.

In summary, with the exception of K'iche' (for which there is reason to doubt the accuracy of – certainly – the frequency counts and – possibly – some semantic features) the discriminative learning model provided a very good fit to both children's and adults' judgment data. As well as simulating an overgeneralization-then-retreat pattern observed for children (e.g., Bowerman, 1988), the model simulated – by virtue of its fine-grained semantic feature representations – children's and adults' ability to judge the acceptability of unfamiliar/novel verbs in particular constructions on the basis of their semantics (e.g., Ambridge et al., 2008).

## 5 General discussion

Explaining how children learn to avoid producing overgeneralization errors such as *The clown laughed/*cried*/*fell*/*disappeared the man*, while retaining the ability to generalize unwitnessed verbs into this construction, has been described as a “learnability paradox” (Pinker, 1989: 415) that represents “one of the most…difficult challenges for all students of language acquisition” (Bowerman, 1988: 73). In hindsight, the findings of the present study suggest not only that there is no paradox, but – on the contrary – that the solution falls naturally out of just about the simplest learning procedure imaginable: a variant of an equation proposed to explain associative learning in rats and mice. When supplied – with realistic relative frequency – with (a) “lexical” identifiers, (b) bundles of human-derived, verb-level semantic features and (c) a crude binary representation of event semantics (causal/non-causal), a simple discriminative learning model was able to predict the relative acceptability of more- and less-transparent causative forms (e.g., *Someone made the man laugh > *Someone laughed the man*) across three age groups (5–6, 9–10, adults) and four languages: English, Hebrew, Hindi and Japanese (and, given the findings of the crosslinguistic semantic analysis, would presumably have done so for K'iche' too, had reliable frequency counts been available).

The implication is that children solve this problem in an analogous way, learning the discriminative validity of particular verb roots (bundles of semantic features picked out by lexical identifiers), in particular meaning contexts (i.e., causal/non-causal), for particular outcome forms (essentially syntactic constructions in English, morphologically-inflected/derived forms in Hebrew, Hindi, Japanese and K'iche'). Particularly key to this account is viewing verb roots as primarily bundles of semantic features (albeit ones with lexical identifiers), which is what allows the model – like human learners – to extend unwitnessed verbs into this construction if, and only if, they have appropriate semantics (e.g., Ambridge et al., 2008).

Previous single-process accounts are not inconsistent with this model; each simply focuses on, or simply labels, a different aspect of the process. Preemption (e.g., Goldberg, 1995) emphasizes children's learning of the discriminative validity of particular verb roots for particular outcome forms in (near-)identical meaning contexts, as in the following formalization from Goldberg (2011: 135), where CxA and CxB are two constructions (e.g, the English periphrastic causative and the English transitive causative):

The probability of CxB statistically preempting CxA for a particular verb, verb_i_ …is equivalent to the probability of CxB, given a discourse context at least as suitable for CxA, and verb_i_.

Entrenchment (Braine & Brooks, 1995) emphasizes children's learning of the discriminative validity of particular verb roots for particular outcome forms, regardless of meaning context. Under the present account, entrenchment-type effects are not absent or irrelevant, but, as learning proceeds, rapidly become less important than preemption-type effects. As soon as the model has learned that, given a causative meaning context, either a more-transparent (e.g., English periphrastic causative) or less-transparent (e.g., English transitive causative) causative form is required (which typically happens by around the fifth epoch), the majority of competition, in a causative meaning context, is between the More- and Less-transparent forms/nodes, with only a minimal amount coming from the Other forms/nodes (which represent non-causal, mainly intransitive forms). Thus although an implication of these findings is that there is no need to draw a sharp distinction between preemption and entrenchment – both simply being effects that fall naturally out of a discriminative learning model – it is certainly true to say that, as a verbal hypothesis (as opposed to a computational model) preemption has far more explanatory power than entrenchment.

The verb-semantics hypothesis (Pinker, 1989; Shibatani & Pardeshi, 2002) emphasizes children's learning the discriminative validity of particular verb roots, *as bundles of semantic features*, in particular meaning contexts (i.e., causal/non-causal), for particular outcome forms. Although Pinker's (1989) specific proposal is based on the notion of discrete semantic classes, the spirit of the proposal – that, distributional information notwithstanding, verb-level semantic properties are the key determinant of form/construction choice – chimes with the present account. This assumption is necessary in order to capture the fact that the model (in the split-half validation test), like children (e.g., Ambridge et al., 2008), is able to use semantics determine the relative acceptability of a verb root *that it has never seen before* in two forms/constructions (e.g., the English periphrastic- versus transitive-causative).

It is important to acknowledge that the model's success relies on the assumption that the competing less- and more-transparent target forms (syntactic constructions for English; morphological constructions for the other four languages) are already known. In reality, of course, learning these forms is not a trivial problem. That said, the entrenchment, preemption and verb-semantics hypotheses also start from the point at which these competing forms are already learned, and seek to explain only how they are subsequently restricted to particular verbs.^7^ Unlike these accounts, however, the present model could in principle be expanded to include this earlier stage of learning by replacing the highly-abstract output language-general representations (Less-Transparent, More-Transparent and Other nodes) with much more detailed, language-specific representations. For example, for an English model, the task could be reframed as learning to sequentially predict the words of a sentence; for (say) a Japanese model as learning to sequentially predict the phonemes of a verb form.

An important advantage, however, of using a very abstract output representation is that the model was able to capture important semantic similarities across languages. A criticism often levelled at Pinker's (1989) account is that it is specific to English, and fails for other languages. While it is no doubt true that the *specific* verb classes that Pinker proposed for English do not apply crosslinguistically, the general principle that verbs that require a more transparent (e.g., periphrastic) causative “have internal causes that would make any external prodding indirect (Pinker, 1989: 302)” has been shown to hold across all five (unrelated) languages studied here (including K'iche' for the three supplementary semantic predictors). Given, in particular, the present finding that semantic ratings obtained by speakers of one language predict patterns of acceptability judgments across the four other languages, there is every reason to believe that this effect of directness of causation, particularly in Shibatani and Pardeshi's (2002) formulation, would extend to at least the other 38 languages which they characterize as having a more- and less-direct causative form. Indeed, we have already begun replicating the present study with Balinese-speaking adults, and preliminary analyses suggest that the present pattern of results holds for this language too.

To sum up, the present study used children's (5–6 years, 9–10 years) and adults' graded acceptability judgments of correct and ungrammatical sentences describing events of causation (e.g., *Someone laughed the man; *Someone made the man laugh*; *Someone broke the truck*; *?Someone made the truck break*), to test the entrenchment, preemption and verb-semantics accounts of how learners come to avoid overgeneralization errors, while retaining the ability to extend unwitnessed verbs into these constructions. Broadly speaking, all three accounts were supported for English, Hebrew, Hindi and Japanese (though less so for K'iche', for which the corpus counts, and possibly semantic ratings, were unreliable) as well as in a collapsed all-languages analysis, and an analysis looking at semantic similarities across languages. We therefore built a discriminative learning model designed to simulate all three effects, which, across verbs, yielded a very good fit to the human judgment data, with correlations of around *r* = 0.75 for previously-seen verbs and *r* = 0.50 in a split-half validation test with previously-unseen verbs.

In conclusion, the model and account that we have developed here, although based around a learning rule that pre-dates the preemption, entrenchment and verb-semantics accounts, represents a significant theoretical advance for the field, as it unifies all three phenomena in a framework that has been shown to explain important phenomena in both human and nonhuman-animal learning.

## CRediT authorship contribution statement

**Ben Ambridge:** Conceptualization, Data curation, Formal analysis, Funding acquisition, Methodology, Project administration, Supervision, Writing - original draft, Writing - review & editing. **Tomoko Tatsumi:** Data curation, Investigation, Methodology, Writing - review & editing. **Laura Doherty:** Data curation, Investigation, Methodology, Writing - review & editing. **Ramya Maitreyee:** Data curation, Investigation, Methodology, Writing - review & editing. **Colin Bannard:** Formal analysis, Writing - review & editing. **Soumitra Samanta:** Investigation, Writing - review & editing. **Stewart McCauley:** Investigation, Writing - review & editing. **Inbal Arnon:** Methodology, Supervision, Writing - review & editing. **Shira Zicherman:** Data curation, Investigation, Methodology, Writing - review & editing. **Dani Bekman:** Investigation, Writing - review & editing. **Amir Efrati:** Investigation, Investigation, Writing - review & editing. **Ruth Berman:** Methodology, Writing - review & editing. **Bhuvana Narasimhan:** Methodology, Writing - review & editing. **Dipti Misra Sharma:** Methodology, Writing - review & editing. **Rukmini Bhaya Nair:** Methodology, Writing - review & editing. **Kumiko Fukumura:** Methodology, Writing - review & editing. **Seth Campbell:** Investigation, Writing - review & editing. **Clifton Pye:** Methodology, Writing - review & editing. **Pedro Mateo Pedro:** Data curation, Methodology, Writing - review & editing. **Sindy Fabiola Can Pixabaj:** Investigation. **Mario Marroquín Pelíz:** Investigation. **Margarita Julajuj Mendoza:** Investigation.

## Figures and Tables

**Figure:**
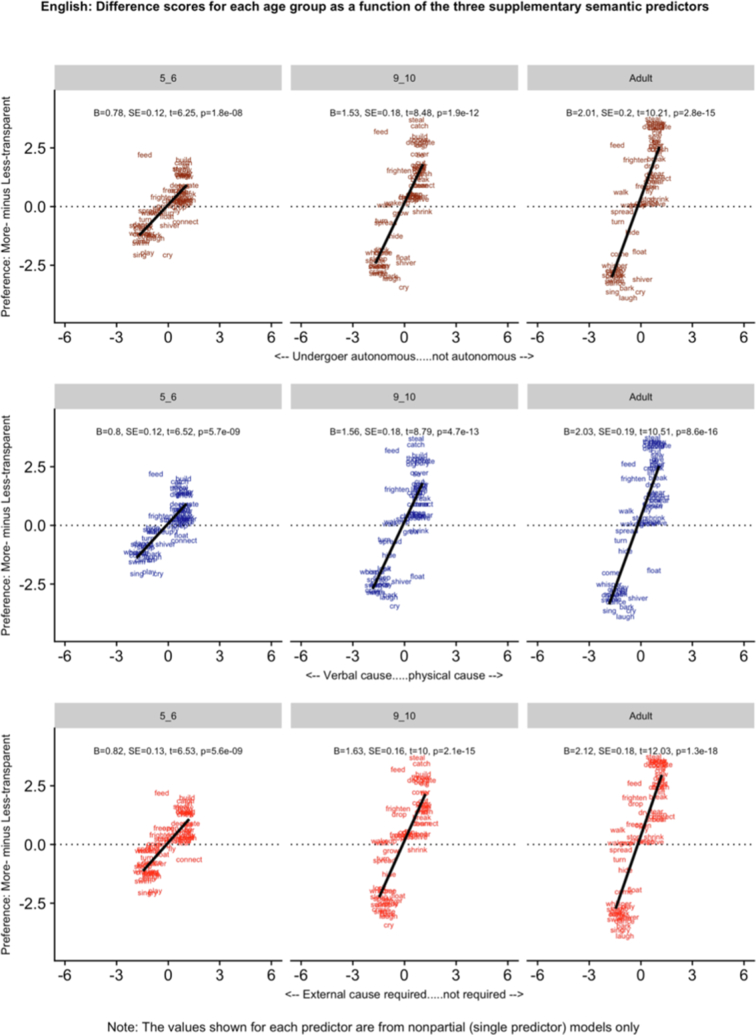


**Figure:**
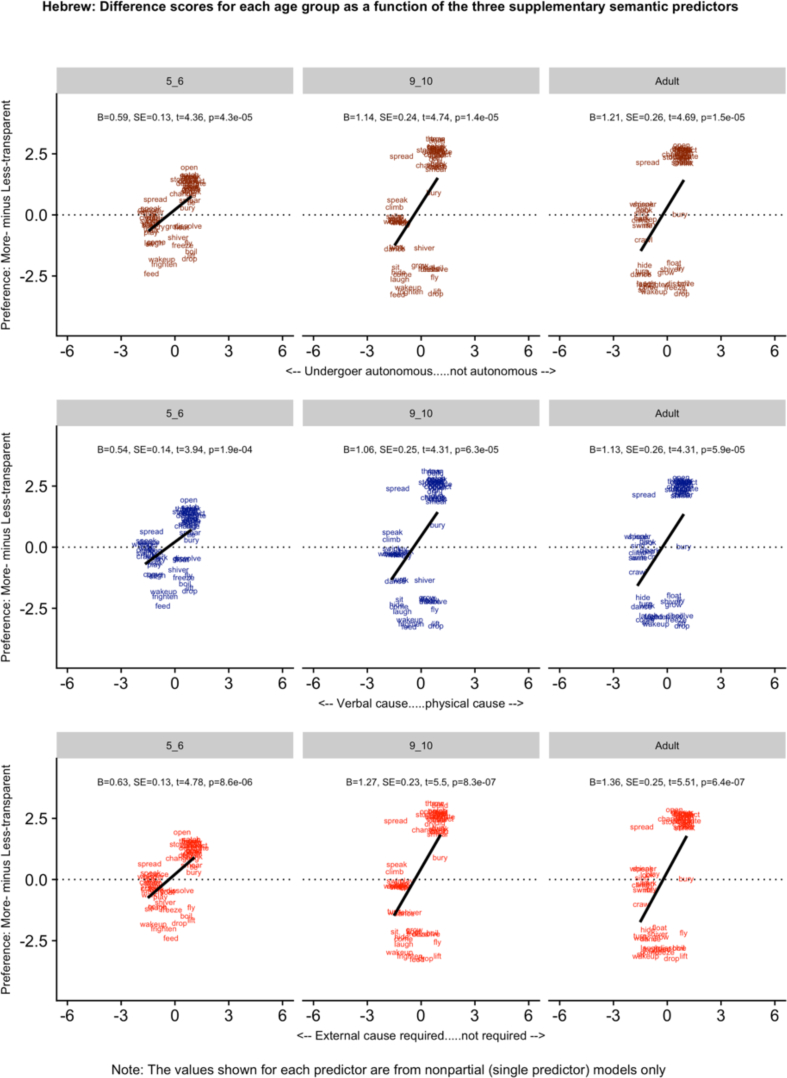


**Figure:**
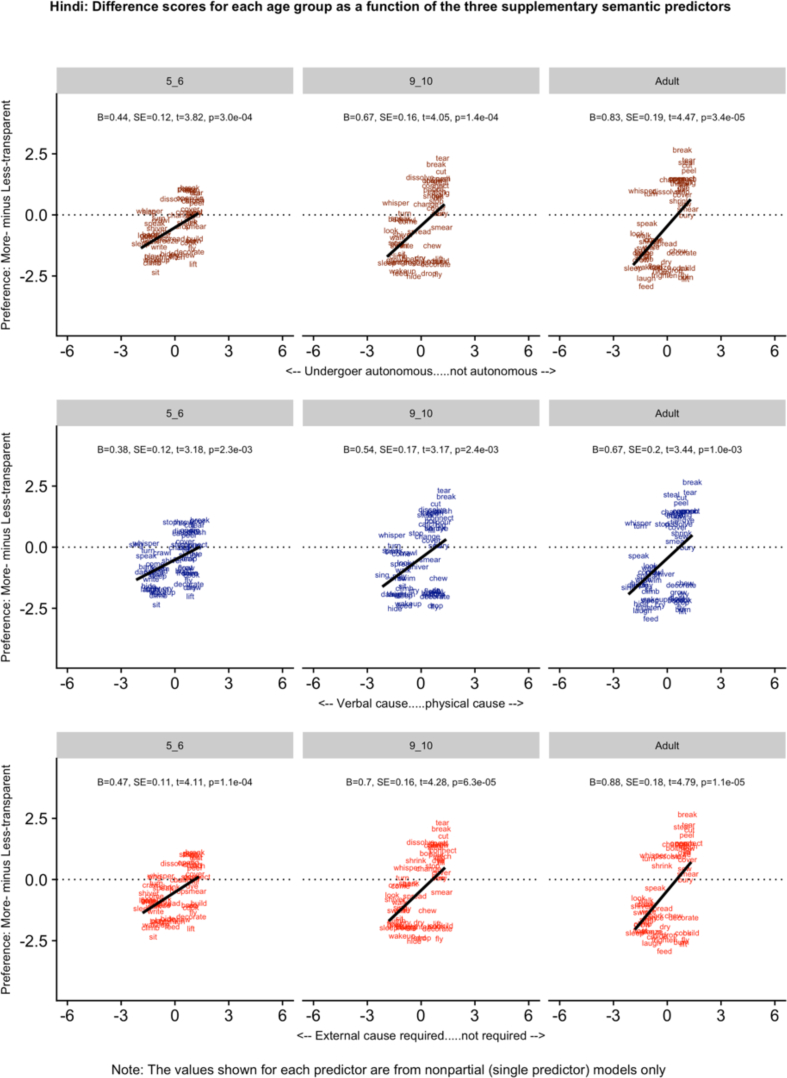


**Figure:**
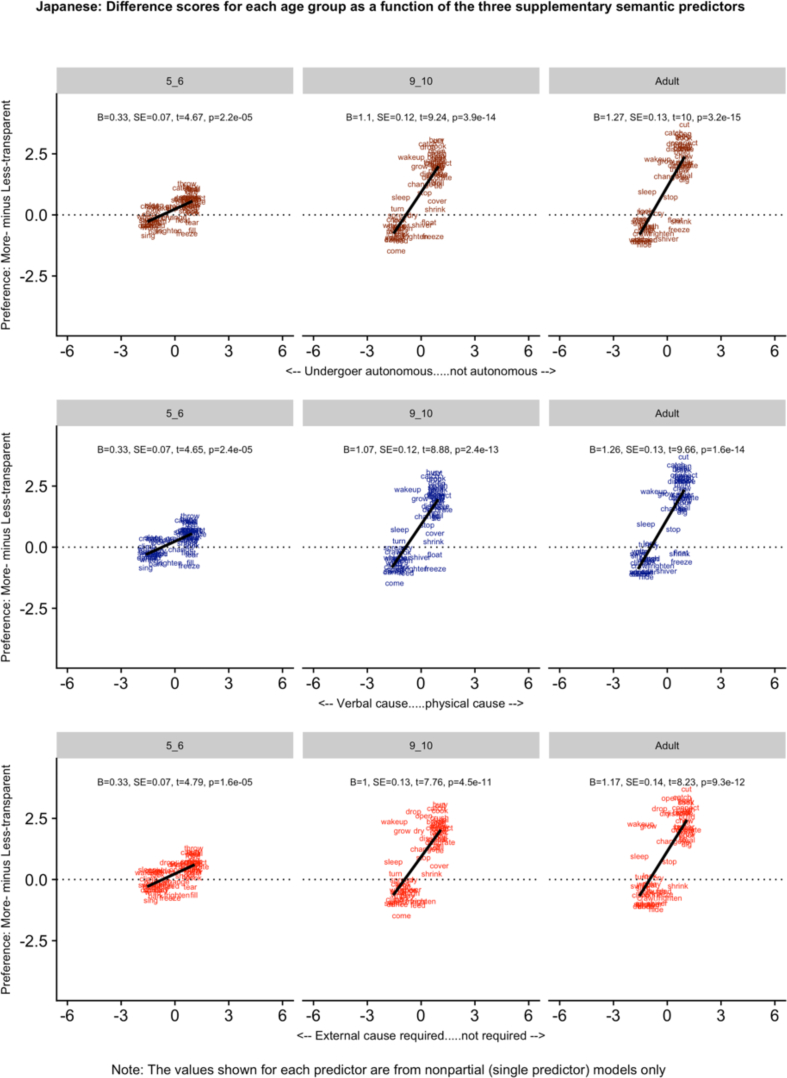


**Figure:**
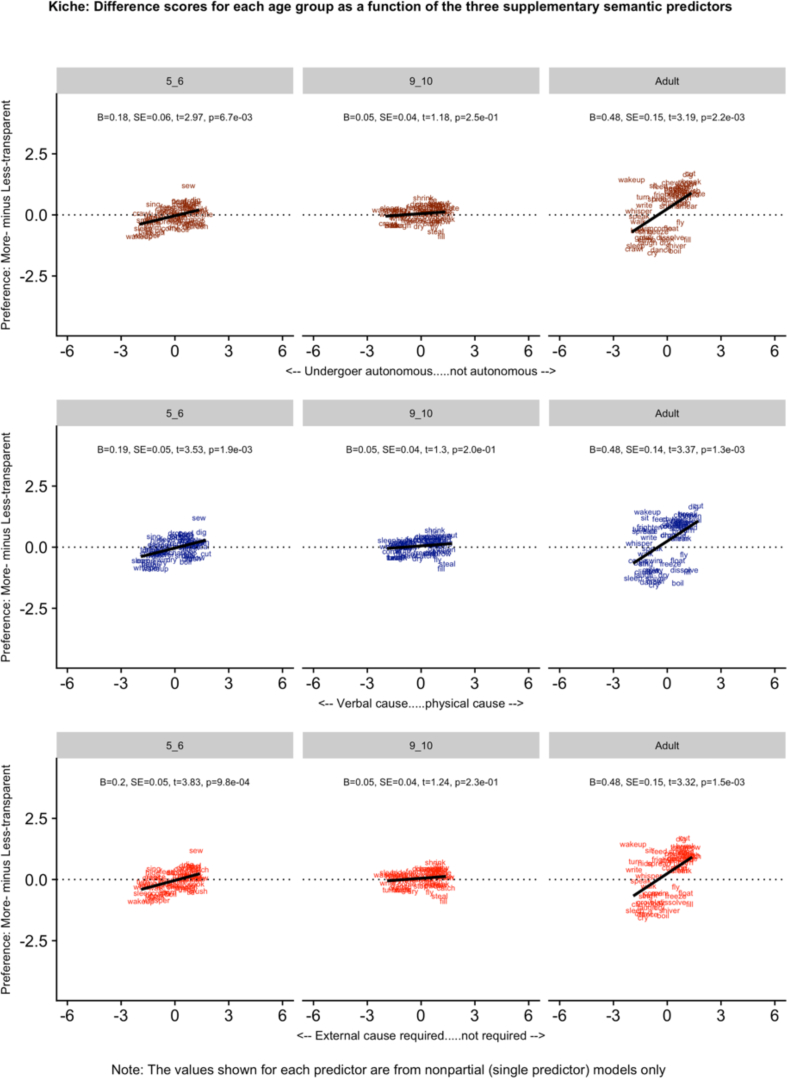


**Figure:**
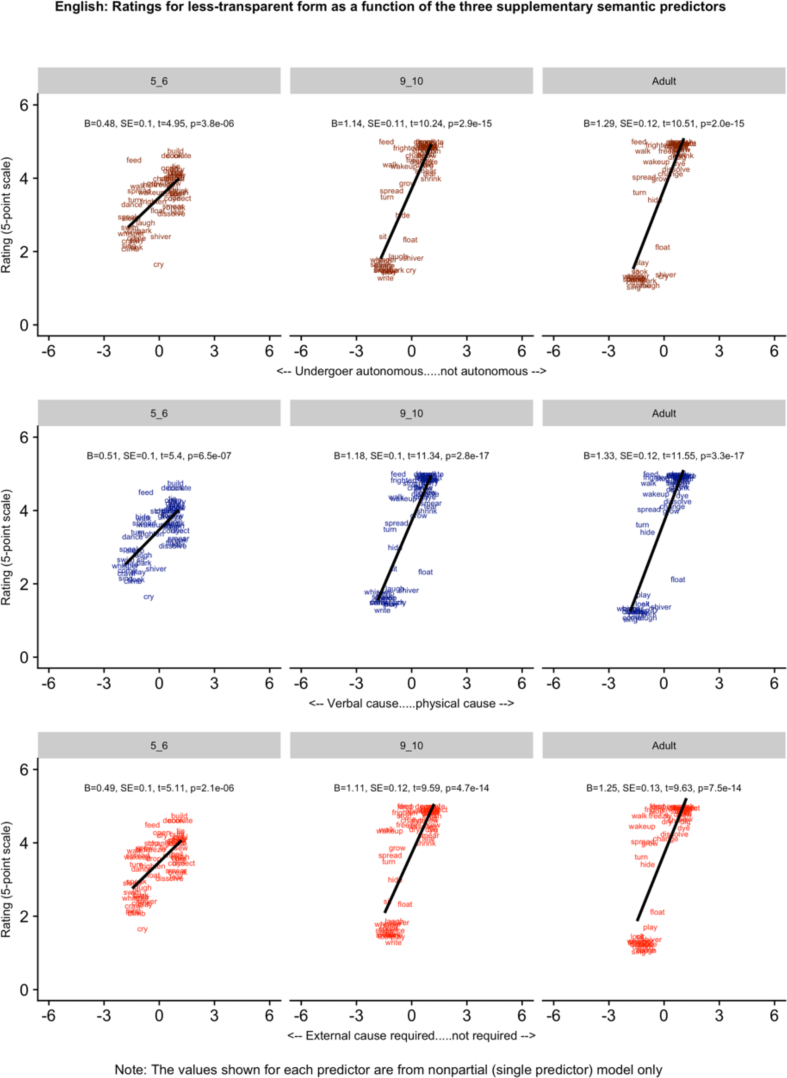


**Figure:**
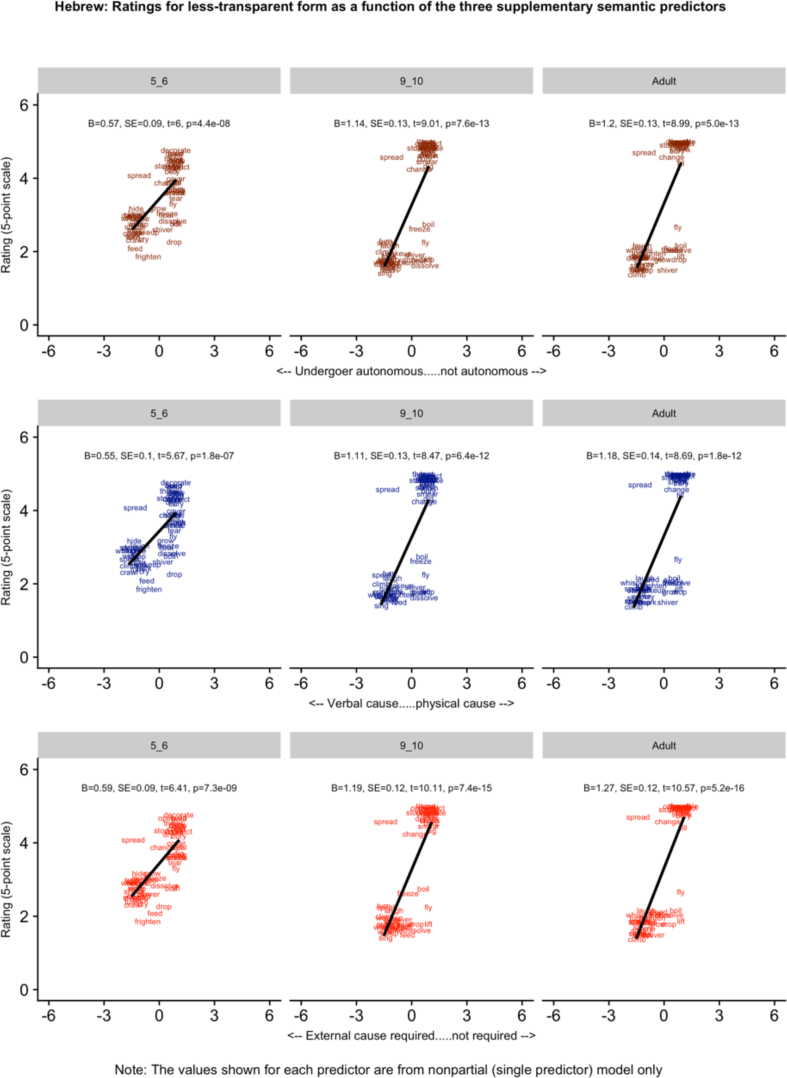


**Figure:**
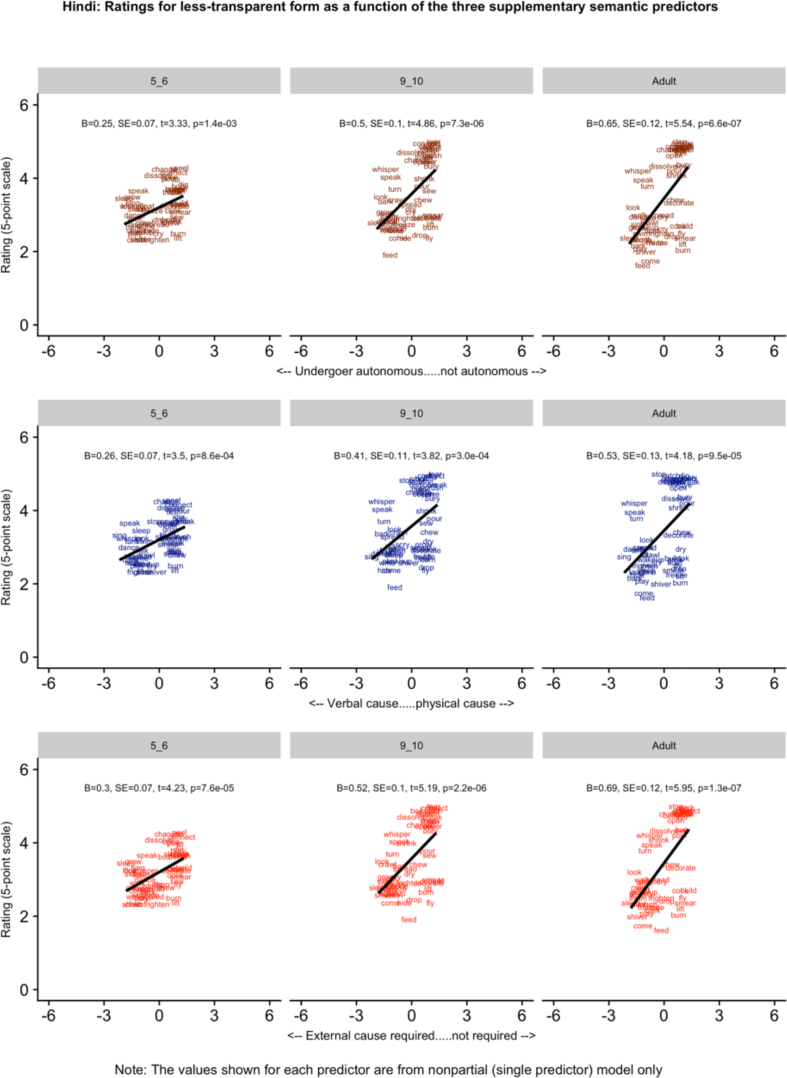


**Figure:**
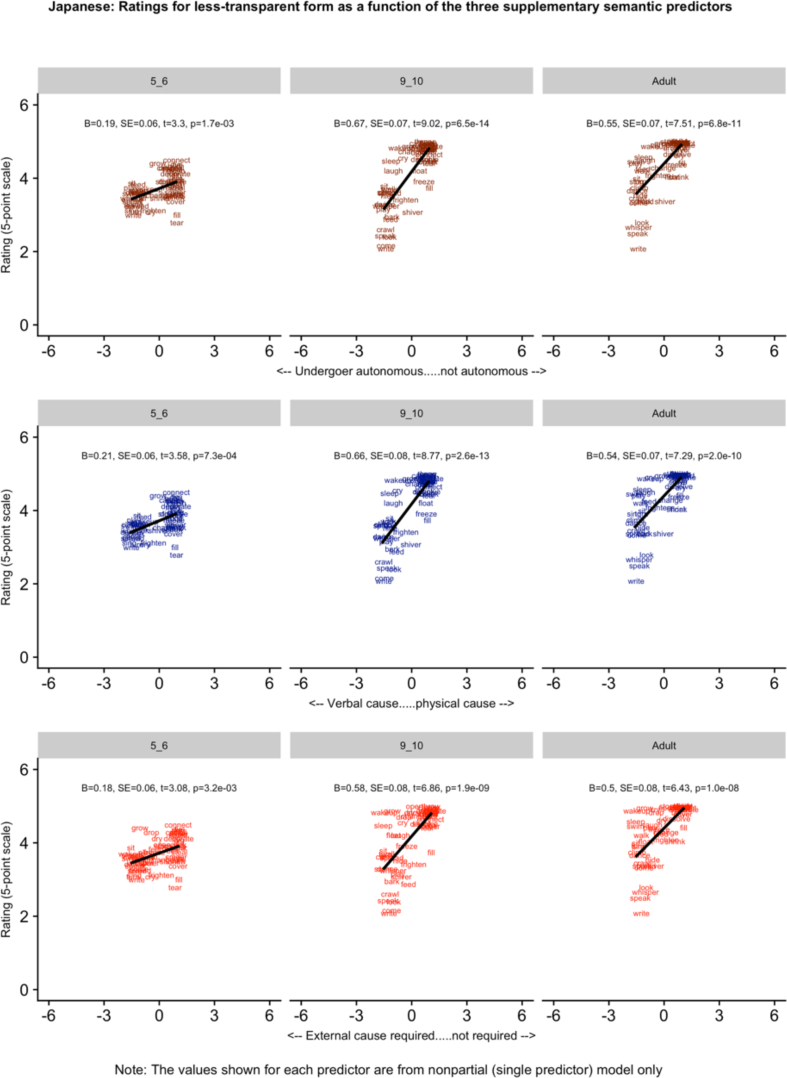


**Figure:**
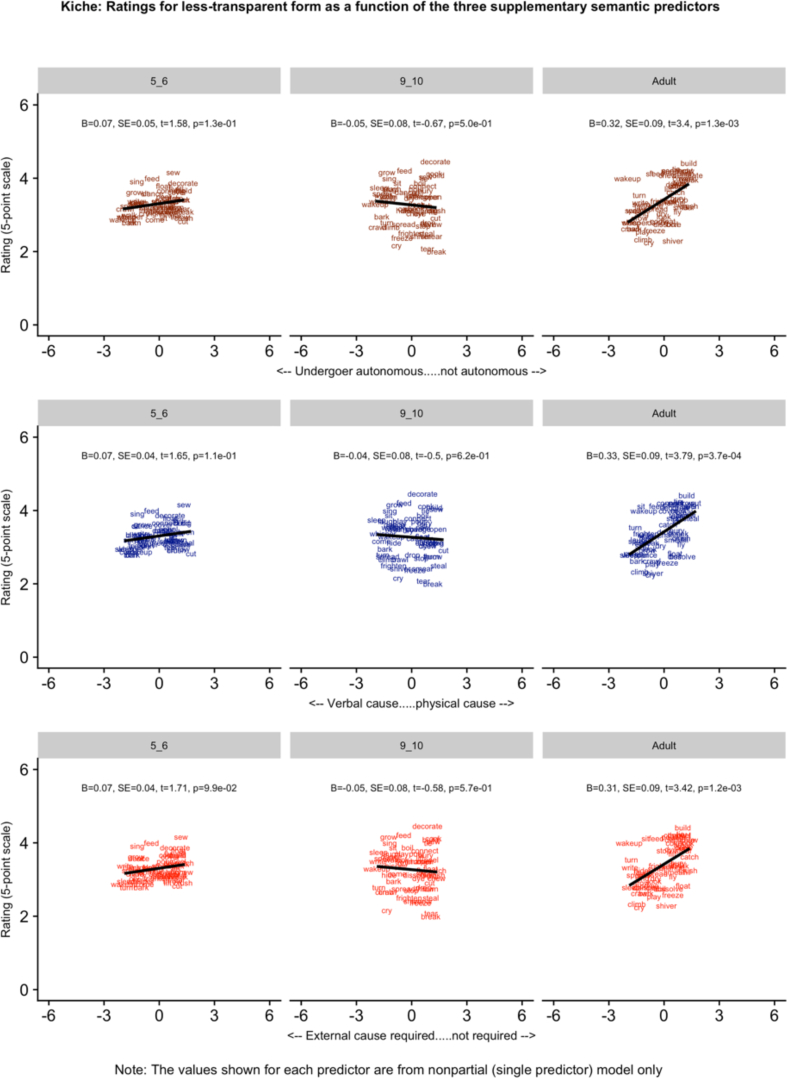


**Figure:**
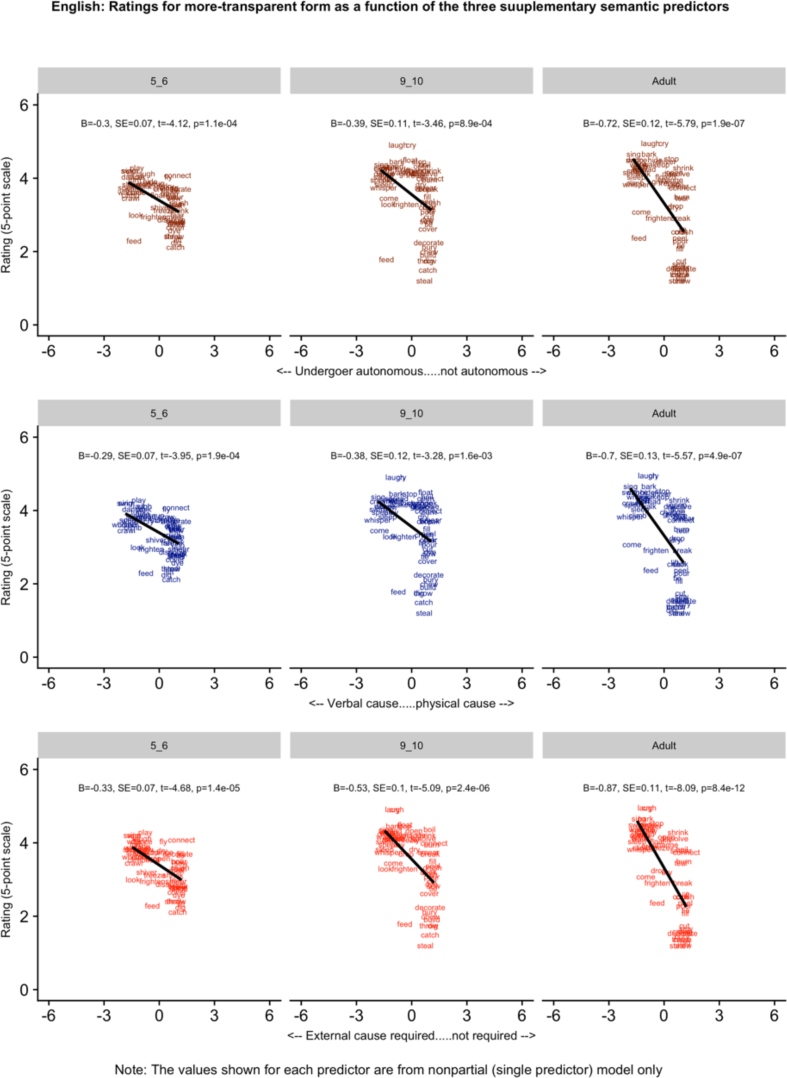


**Figure:**
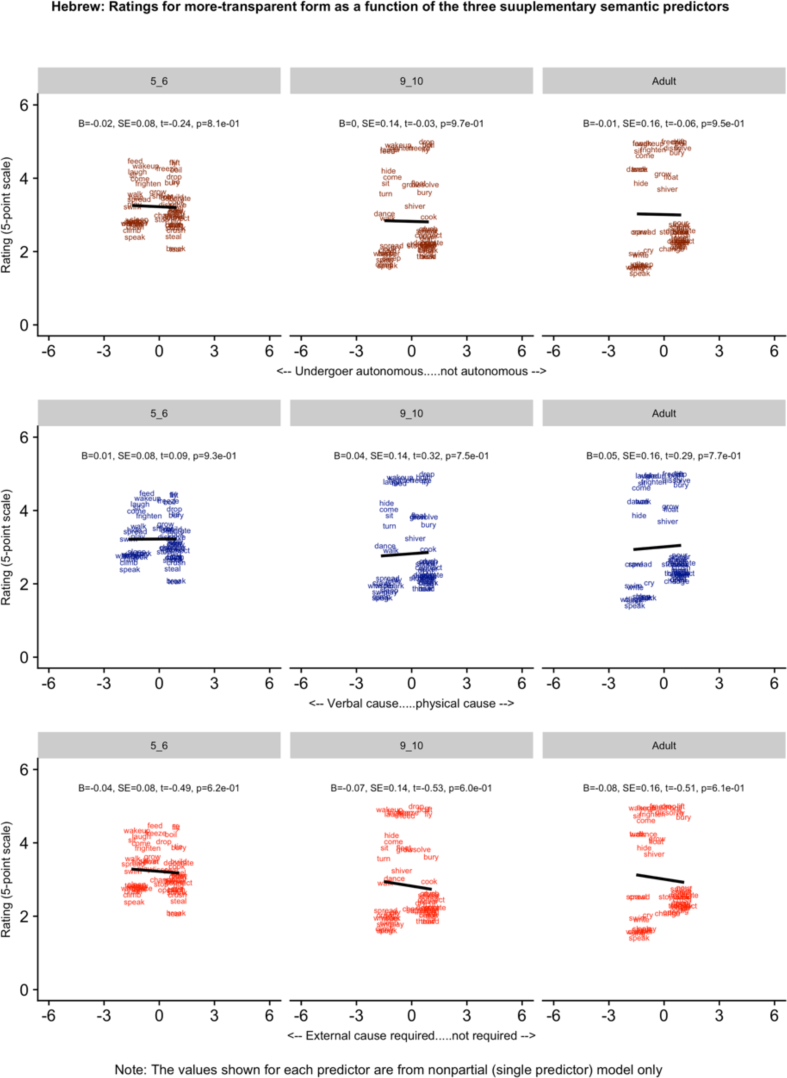


**Figure:**
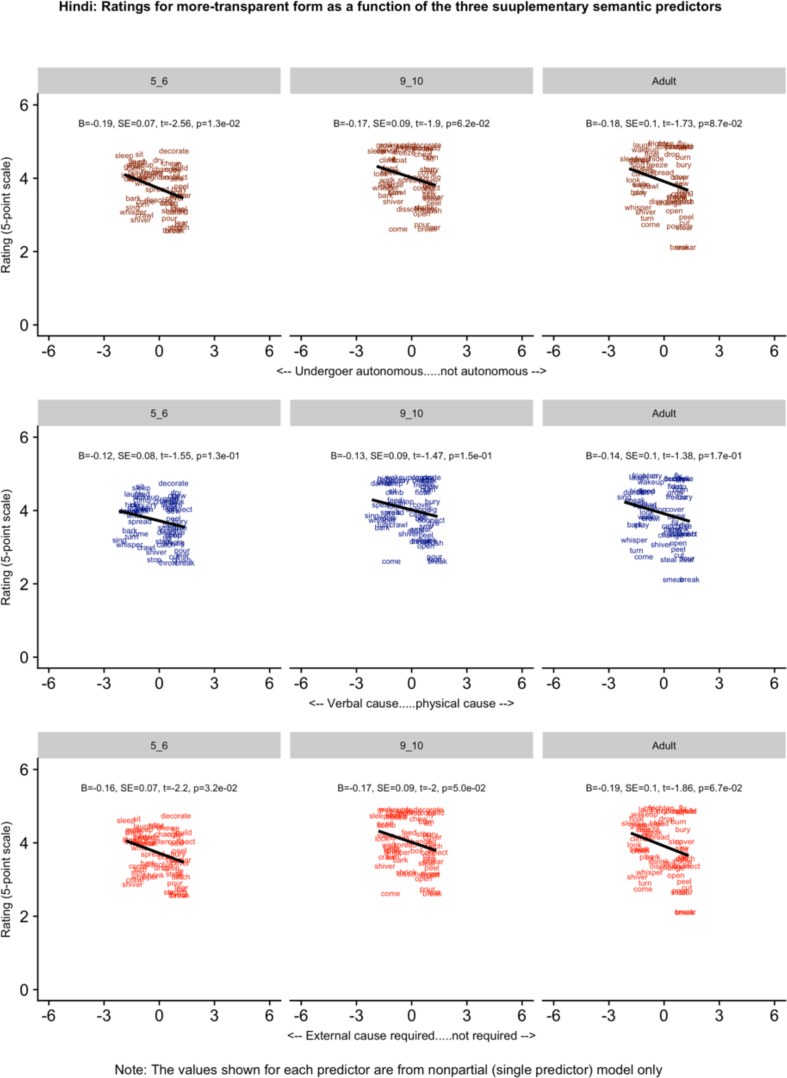


**Figure:**
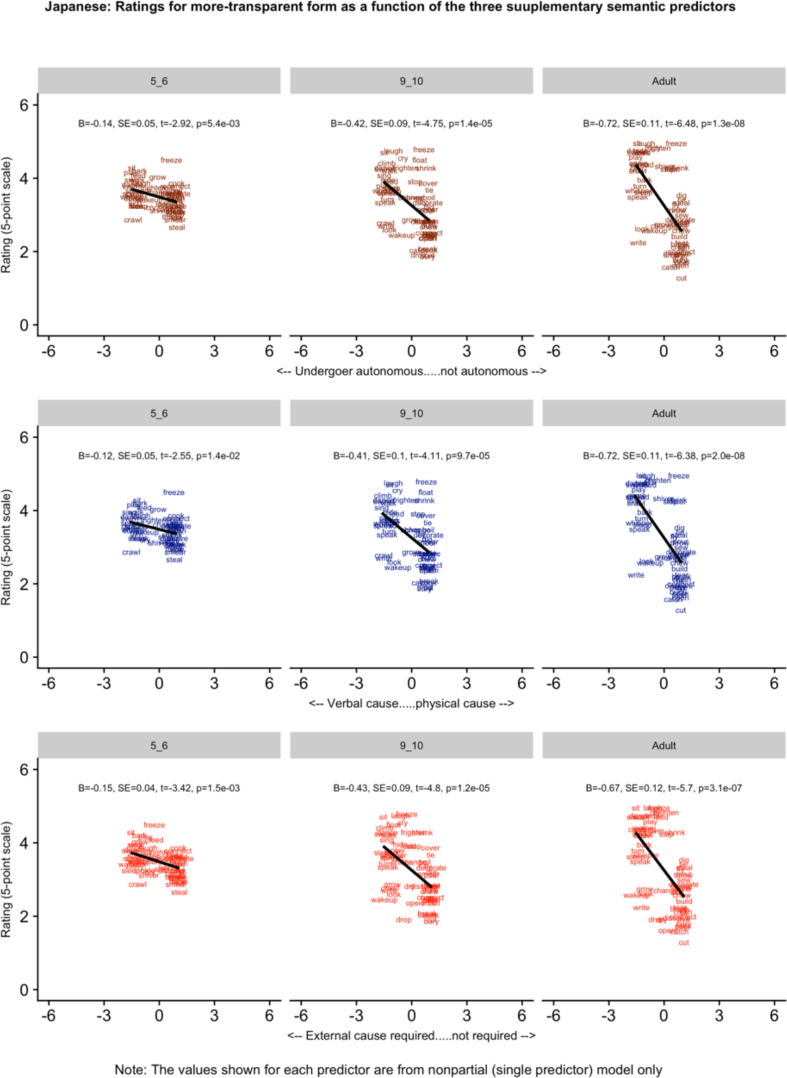


**Figure:**
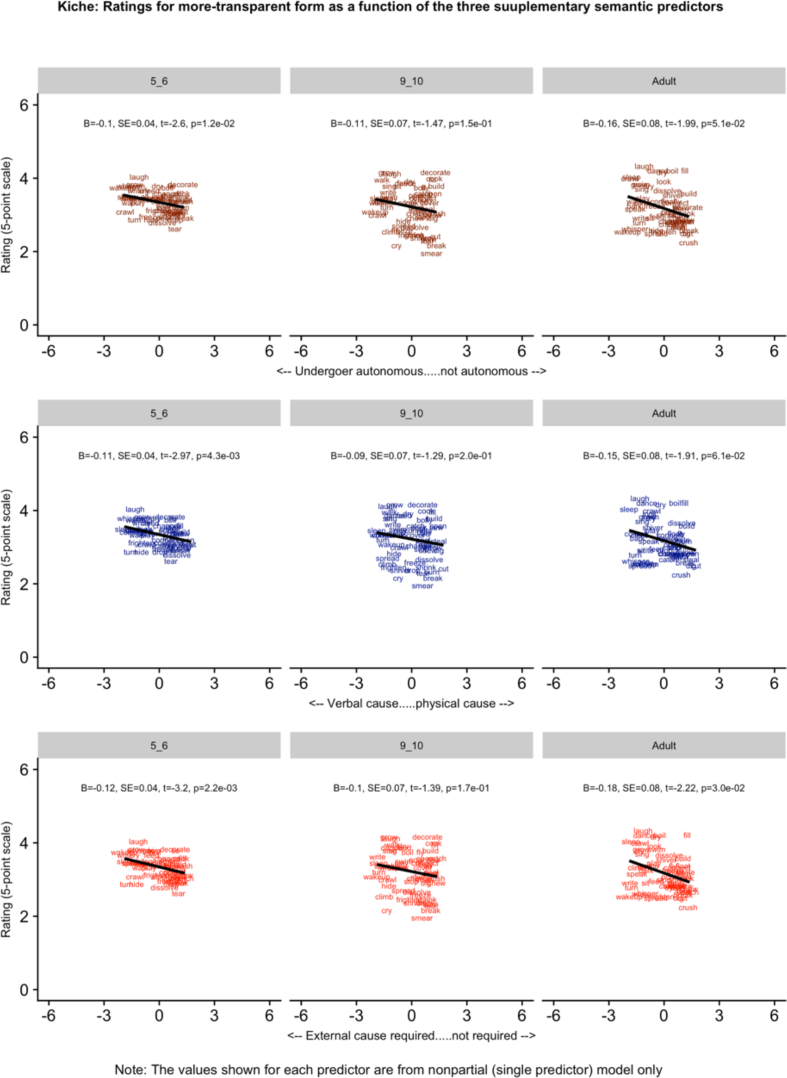


**Table:** 

Difference Scores						
**English**	Preemption	Entrenchment	EventMerge	Autonomy	Directive	Requires
Preemption	1	0.96	0.67	0.65	0.66	0.69
Entrenchment	0.96	1	0.68	0.63	0.63	0.68
EventMerge	0.67	0.68	1	0.9	0.89	0.92
Autonomy	0.65	0.63	0.9	1	0.96	0.93
Directive	0.66	0.63	0.89	0.96	1	0.9
Requires	0.69	0.68	0.92	0.93	0.9	1

**Hebrew**	Preemption	Entrenchment	EventMerge	Autonomy	Directive	Requires
Preemption	1	0.93	0.51	0.5	0.48	0.56
Entrenchment	0.93	1	0.5	0.46	0.45	0.55
EventMerge	0.51	0.5	1	0.87	0.88	0.88
Autonomy	0.5	0.46	0.87	1	0.98	0.95
Directive	0.48	0.45	0.88	0.98	1	0.95
Requires	0.56	0.55	0.88	0.95	0.95	1

**Hindi**	Preemption	Entrenchment	EventMerge	Autonomy	Directive	Requires
Preemption	1	0.94	0.44	0.31	0.27	0.36
Entrenchment	0.94	1	0.34	0.2	0.17	0.23
EventMerge	0.44	0.34	1	0.78	0.67	0.82
Autonomy	0.31	0.2	0.78	1	0.82	0.93
Directive	0.27	0.17	0.67	0.82	1	0.74
Requires	0.36	0.23	0.82	0.93	0.74	1

**Japanese**	Preemption	Entrenchment	EventMerge	Autonomy	Directive	Requires
Preemption	1	0.93	0.58	0.59	0.59	0.55
Entrenchment	0.93	1	0.62	0.63	0.63	0.62
EventMerge	0.58	0.62	1	0.92	0.92	0.91
Autonomy	0.59	0.63	0.92	1	0.99	0.91
Directive	0.59	0.63	0.92	0.99	1	0.89
Requires	0.55	0.62	0.91	0.91	0.89	1

**K'iche**	Preemption	Entrenchment	EventMerge	Autonomy	Directive	Requires
Preemption	1	0.93	0.2	-0.2	-0.2	-0.2
Entrenchment	0.93	1	0.16	-0.1	-0.2	-0.2
EventMerge	0.2	0.16	1	0.35	0.37	0.36
Autonomy	-0.2	-0.1	0.35	1	0.89	0.89
Directive	-0.2	-0.2	0.37	0.89	1	0.91
Requires	-0.2	-0.2	0.36	0.89	0.91	1

**Less-transparent forms**						
**English**	Preemption	Entrenchment	EventMerge	Autonomy	Directive	Requires
Preemption	1	0.93	0.67	0.65	0.66	0.69
Entrenchment	0.93	1	0.74	0.76	0.75	0.75
EventMerge	0.67	0.74	1	0.9	0.89	0.92
Autonomy	0.65	0.76	0.9	1	0.96	0.93
Directive	0.66	0.75	0.89	0.96	1	0.9
Requires	0.69	0.75	0.92	0.93	0.9	1

**Hebrew**	Preemption	Entrenchment	EventMerge	Autonomy	Directive	Requires
Preemption	1	0.86	0.51	0.5	0.48	0.56
Entrenchment	0.86	1	0.77	0.77	0.75	0.81
EventMerge	0.51	0.77	1	0.87	0.88	0.88
Autonomy	0.5	0.77	0.87	1	0.98	0.95
Directive	0.48	0.75	0.88	0.98	1	0.95
Requires	0.56	0.81	0.88	0.95	0.95	1
	
**Hindi**	Preemption	Entrenchment	EventMerge	Autonomy	Directive	Requires
Preemption	1	0.89	0.44	0.31	0.27	0.36
Entrenchment	0.89	1	0.53	0.43	0.4	0.46
EventMerge	0.44	0.53	1	0.78	0.67	0.82
Autonomy	0.31	0.43	0.78	1	0.82	0.93
Directive	0.27	0.4	0.67	0.82	1	0.74
Requires	0.36	0.46	0.82	0.93	0.74	1

**Japanese**	Preemption	Entrenchment	EventMerge	Autonomy	Directive	Requires
Preemption	1	0.76	0.58	0.59	0.59	0.55
Entrenchment	0.76	1	0.6	0.67	0.66	0.67
EventMerge	0.58	0.6	1	0.92	0.92	0.91
Autonomy	0.59	0.67	0.92	1	0.99	0.91
Directive	0.59	0.66	0.92	0.99	1	0.89
Requires	0.55	0.67	0.91	0.91	0.89	1

**K'iche**	Preemption	Entrenchment	EventMerge	Autonomy	Directive	Requires
Preemption	1	0.75	0.2	-0.2	-0.2	-0.2
Entrenchment	0.75	1	0.09	-0.2	-0.2	-0.2
EventMerge	0.2	0.09	1	0.35	0.37	0.36
Autonomy	-0.2	-0.2	0.35	1	0.89	0.89
Directive	-0.2	-0.2	0.37	0.89	1	0.91
Requires	-0.2	-0.2	0.36	0.89	0.91	1

**More-transparent forms**						
**English**	Preemption	Entrenchment	EventMerge	Autonomy	Directive	Requires
Preemption	1	0.69	0.67	0.65	0.66	0.69
Entrenchment	0.69	1	0.35	0.23	0.24	0.35
EventMerge	0.67	0.35	1	0.9	0.89	0.92
Autonomy	0.65	0.23	0.9	1	0.96	0.93
Directive	0.66	0.24	0.89	0.96	1	0.9
Requires	0.69	0.35	0.92	0.93	0.9	1

**Hebrew**	Preemption	Entrenchment	EventMerge	Autonomy	Directive	Requires
Preemption	1	0.5	0.51	0.5	0.48	0.56
Entrenchment	0.5	1	-0.1	-0.2	-0.2	-0.1
EventMerge	0.51	-0.1	1	0.87	0.88	0.88
Autonomy	0.5	-0.2	0.87	1	0.98	0.95
Directive	0.48	-0.2	0.88	0.98	1	0.95
Requires	0.56	-0.1	0.88	0.95	0.95	1

**Hindi**	Preemption	Entrenchment	EventMerge	Autonomy	Directive	Requires
Preemption	1	0.44	0.44	0.31	0.27	0.36
Entrenchment	0.44	1	-0.1	-0.2	-0.2	-0.2
EventMerge	0.44	-0.1	1	0.78	0.67	0.82
Autonomy	0.31	-0.2	0.78	1	0.82	0.93
Directive	0.27	-0.2	0.67	0.82	1	0.74
Requires	0.36	-0.2	0.82	0.93	0.74	1

**Japanese**	Preemption	Entrenchment	EventMerge	Autonomy	Directive	Requires
Preemption	1	0.64	0.58	0.59	0.59	0.55
Entrenchment	0.64	1	0.28	0.19	0.21	0.17
EventMerge	0.58	0.28	1	0.92	0.92	0.91
Autonomy	0.59	0.19	0.92	1	0.99	0.91
Directive	0.59	0.21	0.92	0.99	1	0.89
Requires	0.55	0.17	0.91	0.91	0.89	1

**K'iche**	Preemption	Entrenchment	EventMerge	Autonomy	Directive	Requires
Preemption	1	0.53	0.2	-0.2	-0.2	-0.2
Entrenchment	0.53	1	0.12	-0	-0.1	-0.1
EventMerge	0.2	0.12	1	0.35	0.37	0.36
Autonomy	-0.2	-0	0.35	1	0.89	0.89
Directive	-0.2	-0.1	0.37	0.89	1	0.91
Requires	-0.2	-0.1	0.36	0.89	0.91	1

## Appendix 1 Verb-guessing pilot

Spreadsheet (.csv) files summarizing these data can be downloaded from https://osf.io/pavm7/

## Appendix 2 Supplementary semantic ratings task instructions

**Autonomy**

Here, A (THE UNSEEN-CAUSER) causes B (the PERSON/THING on the stage) to carry out/undergo an ACTION/EVENT/CHANGE. We are interested in how much choice B has over the ACTION/EVENT/CHANGE. Please rate the extent to which.

[Left anchor] B chooses to carry out/undergo the ACTION/EVENT/CHANGE

[Right anchor] A forces B to carry out/undergo the ACTION/EVENT/CHANGE, whether B likes it or not.

**Directive**

Here, A (THE UNSEEN-CAUSER) causes B (the PERSON/THING on the stage)to carry out/undergo an ACTION/EVENT/CHANGE. We are interested in how A would usually cause this type ACTION/EVENT/CHANGE. Please rate the extent to which."

[Left anchor] A can only ask/tell/instruct B to undergo the ACTION/EVENT/CHANGE

[Right anchor] A can ensure that the ACTION/EVENT/CHANGE happens by physically manipulating B.

**Requires**

"Here, A (THE UNSEEN-CAUSER) causes B (the PERSON/THING on the stage)to carry out/undergo an ACTION/EVENT/CHANGE. We are interested in the extent to which A is necessary in bringing about this ACTION/EVENT/CHANGE. Please rate the extent to which."

B's ACTION/EVENT/CHANGE could come about with absolutely no involvement from A

B's ACTION/EVENT/CHANGE requires A's involvement, and could not happen without A

## Appendix 7 Simultaneous models with Event Merge semantics plus (a) preemption only, (b) entrenchment only

**Extra simultaneous models: Preemption + Event Merge**

English Adults: *B=*1.19, *SE=*0.2, *t=*5.92, *p=*1.7e-07

English 9-10 Year olds: *B=*1, *SE=*0.18, *t=*5.72, *p=*3.3e-07

English 5-6 Year olds: *B=*0.53, *SE=*0.13, *t=*4.07, *p=*1.2e-04

Hebrew Adults: *B=*2, *SE=*0.15, *t=*13.02, *p=*4.5e-22

Hebrew 9-10 Year olds: *B=*1.88, *SE=*0.14, *t=*13.11, *p=*1.0e-20

Hebrew 5-6 Year olds: *B=*0.89, *SE=*0.12, *t=*7.23, *p=*2.4e-10

Hindi Adults: *B=*0.88, *SE=*0.16, *t=*5.6, *p=*5.0e-07

Hindi 9-10 Year olds: *B=*0.7, *SE=*0.14, *t=*4.89, *p=*7.3e-06

Hindi 5-6 Year olds: *B=*0.46, *SE=*0.09, *t=*5.02, *p=*6.3e-06

Japanese Adults: *B=*0.56, *SE=*0.14, *t=*4.14, *p=*1.0e-04

Japanese 9-10 Year olds: *B=*0.69, *SE=*0.11, *t=*6.27, *p=*2.5e-08

Japanese 5-6 Year olds: *B=*0.22, *SE=*0.08, *t=*2.69, *p=*9.5e-03

K’iche’ Adults: *B=*-0.26, *SE=*0.13, *t=*-2.03, *p=*4.7e-02

K’iche’ 9-10 Year olds: *B=*-0.01, *SE=*0.05, *t=*-0.16, *p=*8.8e-01

K’iche’ 5-6 Year olds: *B=*-0.1, *SE=*0.06, *t=*-1.64, *p=*1.1e-01

**Extra simultaneous models: Entrenchment + Event Merge**

English Adults: *B=*1.19, *SE=*0.2, *t=*5.9, *p=*1.9e-07

English 9-10 Year olds: *B=*1.03, *SE=*0.18, *t=*5.91, *p=*1.5e-07

English 5-6 Year olds: *B=*0.53, *SE=*0.13, *t=*4.07, *p=*1.2e-04

Hebrew Adults: *B=*1.9, *SE=*0.17, *t=*11.02, *p=*1.0e-17

Hebrew 9-10 Year olds: *B=*1.79, *SE=*0.16, *t=*11.26, *p=*2.4e-17

Hebrew 5-6 Year olds: *B=*0.87, *SE=*0.13, *t=*6.94, *p=*8.0e-10

Hindi Adults: *B=*0.88, *SE=*0.15, *t=*5.78, *p=*2.3e-07

Hindi 9-10 Year olds: *B=*0.72, *SE=*0.14, *t=*5.27, *p=*1.7e-06

Hindi 5-6 Year olds: *B=*0.47, *SE=*0.09, *t=*5.37, *p=*1.8e-06

Japanese Adults: *B=*0.51, *SE=*0.15, *t=*3.5, *p=*8.5e-04

Japanese 9-10 Year olds: *B=*0.59, *SE=*0.12, *t=*4.79, *p=*1.0e-05

Japanese 5-6 Year olds: *B=*0.22, *SE=*0.08, *t=*2.7, *p=*9.1e-03

K’iche’ Adults: *B=*-0.19, *SE=*0.13, *t=*-1.54, *p=*1.3e-01

K’iche’ 9-10 Year olds: *B=*0, *SE=*0.05, *t=*0, *p=*1.0e+00

K’iche’ 5-6 Year olds: *B=*-0.09, *SE=*0.06, *t=*-1.52, *p=*1.4e-01

## Footnotes

1 Ahtna, Alamblak, Amharic, Asheninka, Bella Coola, Burmese, Cantonese, Carrier, Creek, English, French, German, Guaraní, Hualapai, Hungarian, Japanese, Kammu, Kinyarwanda, Korean, Koyukon, Lushai, Malay, Manchu, Mandarin, Marathi, Matses, Navajo, Olutec, Quechua, Shipibo-Konibo, Sikuani, Svan, Tarascan, Tiddim Chin, Turkish, Yidiny, Yimas and Yukaghir.

2 As a reviewer noted, this general idea is quite old, dating back at least to Fodor (1970), Givon (1979) and Hopper and Thompson (1980).

3 For the sake of clarity, we should emphasize that what we are calling simply *entrenchment* is what Stefanowitsch (2008) calls *negative entrenchment* and what Goldberg (2019) calls *conservatism* via *entrenchment*. That is, we use entrenchment to refer an inference from absence, and not – as its in original sense (Langacker, 1988) – to simple frequency.

4 The original preregistered version mistakenly said “100 semantic raters”. It was never our intention to aggregate the semantic ratings across language (as this original figure suggests), but in the present version we explore crosslinguistic semantic effects using additional exploratory analyses.

5 The following by-participant random slopes had to be removed in order to enable the models to converge: (a) English 9–10-year-olds single-predictor Event-Merge model for difference scores: Event-Merge. (b) English 9–10-year-olds simultaneous model for less-transparent forms: Preemption. (c) English 9–10-year-olds single-predictor Preemption model for more-transparent forms: Preemption. (d) English 9–10-year-olds simultaneous model for more-transparent forms: Entrenchment. (e) Japanese 9–10-year-olds single-predictor Autonomy model for more-transparent forms: Autonomy. (f) Japanese 9–10-year-olds single-predictor Requires model for more-transparent forms: Requires. (g) Hebrew 9–10-year-olds single-predictor Preemption model for less-transparent forms: Preemption. (h) Hebrew 9–10-year-olds simultaneous model for less-transparent forms: Event-Merge.

6 Although the languages in the present study are mainly from different families, English and Hindi are (quite distantly) related at the level of Proto-Indo-European.

7 Another model with this property is Yang's sufficiency/tolerance principle account, as applied to the English causative constructions by Irani (2009) and to the Korean causative constructions by Lee and Kodner (2019). The assumption here is that learners store a rule (e.g., for forming the less transparent causative) and a list of exceptions (i.e., verbs that cannot grammaticality appear in this construction, and must instead take the more transparent causative). Although, this account explains aspects of children's naturalistic speech data and, for Korean, binary judgment data, it is difficult to see how a non-probabilistic account based on rules and exceptions can explain the continuous cline of acceptability judgments found in the present studies; less still why this cline is so well predicted by a model that has access to distributional and semantic information, but not the sufficiency or tolerance principle.
